# Regulatory T cells decrease C3-positive reactive astrocytes in Alzheimer-like pathology

**DOI:** 10.1186/s12974-023-02702-3

**Published:** 2023-03-08

**Authors:** Grégoire Stym-Popper, Karen Matta, Thomas Chaigneau, Roshan Rupra, Alexandros Demetriou, Stéphane Fouquet, Cira Dansokho, Cécile Toly-Ndour, Guillaume Dorothée

**Affiliations:** 1 Sorbonne Université, INSERM UMRS 938, Centre de Recherche Saint-Antoine, CRSA, Immune System and Neuroinflammation Laboratory, Hôpital Saint-Antoine, 184 rue du Faubourg Saint-Antoine, 75012 Paris, France; 2 University of Southampton, University Hospital Southampton NHS Foundation Trust, Southampton, UK; 3 Imaging Platform, Sorbonne Université, INSERM UMR_S968 and CNRS UMR7210, Institut de la Vision, 75012 Paris, France; 4 Present Address: Evotec SE, Manfred Eugen Campus, Hamburg, Germany; 5 Present Address: Sorbonne Université, APHP, Laboratoire du Centre National de Référence en Hémobiologie Périnatale-DMU Biogem, Hôpital St Antoine, Paris, France

**Keywords:** Astrocytes, Regulatory T cells, Neuroinflammation, Alzheimer’s disease

## Abstract

**Background:**

Increasing evidence supports a key role for peripheral immune processes in the pathophysiology of Alzheimer’s disease (AD), highlighting an intricate interplay between brain resident glial cells and both innate and adaptive peripheral immune effectors. We previously showed that regulatory T cells (Tregs) have a beneficial impact on disease progression in AD-like pathology, notably by modulating the microglial response associated with Aβ deposits in a mouse model of amyloid pathology. Besides microglia, reactive astrocytes also play a critical role in neuroinflammatory processes associated with AD. Different phenotypes of reactive astrocytes have previously been characterized, including A1-like neurotoxic and A2-like neuroprotective subtypes. However, the precise impact of Tregs on astrocyte reactivity and phenotypes in AD still remains poorly defined.

**Methods:**

We assessed the impact of Treg immunomodulation on astrocyte reactivity in a mouse model of AD-like amyloid pathology. Using 3D imaging, we carried out extensive morphological analyses of astrocytes following either depletion or amplification of Tregs. We further assessed the expression of several A1- and A2-like markers by immunofluorescence and RT-qPCR.

**Results:**

Modulation of Tregs did not significantly impact the magnitude of global astrocyte reactivity in the brain nor in the close vicinity of cortical amyloid deposits. We did not observe changes in the number, morphology, or branching complexity of astrocytes according to immunomodulation of Tregs. However, early transient depletion of Tregs modulated the balance of reactive astrocyte subtypes, resulting in increased C3-positive A1-like phenotypes associated with amyloid deposits. Conversely, early depletion of Tregs decreased markers of A2-like phenotypes of reactive astrocytes associated with larger amyloid deposits. Intriguingly, modulation of Tregs also impacted the cerebral expression of several markers of A1-like subsets in healthy mice.

**Conclusions:**

Our study suggests that Tregs contribute to modulate and fine-tune the balance of reactive astrocyte subtypes in AD-like amyloid pathology, by dampening C3-positive astrocytes in favor of A2-like phenotypes. This effect of Tregs may partly relate to their capacity at modulating steady state astrocyte reactivity and homeostasis. Our data further highlight the need for refined markers of astrocytes subsets and strategy of analysis for better deciphering the complexity of astrocyte reactivity in neurodegeneration.

**Supplementary Information:**

The online version contains supplementary material available at 10.1186/s12974-023-02702-3.

## Background

Alzheimer’s disease (AD) is a neurodegenerative disorder characterized by progressive loss of memory and cognitive functions. Neuropathological features include extracellular deposits of amyloid-β (Aβ) peptide, neurofibrillary tangles consisting of intraneuronal fibrillar aggregates of hyperphosphorylated Tau proteins, and chronic neuroinflammation mediated by activated microglia and reactive astrocytes. Strong accumulating evidence support that chronic innate neuroinflammation plays a complex role in the disease pathophysiology, with both beneficial and detrimental effects [1]. In AD, several populations of activated microglial cells have been described and associated with either aging and/or disease progression, although their relative roles in disease pathogenesis are still a matter of debate [2–4]. Besides their key contribution to the formation and maintenance of the blood–brain barrier, astrocytes play a critical role in regulating neuronal homeostasis, notably by modulating synaptogenesis, neuronal differentiation, neuronal survival and synaptic transmission. Their functional properties include the secretion of neurotrophic factors, release and clearance of neurotransmitters, and regulation of extracellular ion concentration [5, 6]. Similar to microglia, astrocytes become reactive upon activation by misfolded pathogenic proteins, such as Aβ and pathological Tau species. Growing evidence suggests an instrumental role of reactive astrocytes in the pathogenesis of neurodegeneration, e.g. through the production of pro-inflammatory neurotoxic factors, altered phagocytic activity and far-reaching effects on synaptic connectivity [6, 7]**.** Astrocyte reactivity translates into modified cell morphology, changes in gene expression patterns and shifts in their physiological functions [8, 9]. By analogy to microglia, different functional subtypes of reactive astrocytes termed A1 and A2 have been previously described [10, 11]. Whereas some previous evidence suggested a potential beneficial impact of reactive astrocytes in Alzheimer’s disease [12], recent studies support an enrichment of C3^+^ potentially pro-inflammatory neurotoxic A1-like reactive astrocytes in a mouse model of AD-like amyloid pathology [13] and in post-mortem tissues from patients with AD [10]. Increased levels of A1-derived complement molecules were further evidenced in astrocyte-derived exosomes of patients with AD [14].

Besides innate neuroinflammation, accumulating neuropathological, preclinical and genetic data now emphasize an instrumental role of cellular adaptive immunity in Alzheimer’s disease, although its impact on disease progression remains ill-defined. Previous studies suggest a complex implication of peripheral T cell immunity in the pathophysiology of the disease, with either detrimental or beneficial outcomes that may depend on the relative magnitude and functionality of various T cell subsets and/or responses. Whereas some works in mouse models of amyloid pathology reported that Aβ-specific CD8^+^ T cells do not trigger detrimental autoimmune neuroinflammation [15], other reports suggested that different subtypes of Aβ-reactive CD4^+^ T cells may promote either meningoencephalitis and Aβ clearance [16], increased Aβ deposition and microglial activation with worsened cognition [17], or decreased synaptic loss and improved memory [18, 19]. Recent studies in a murine model of AD-like Tau pathology evidenced that peripheral T cell depletion dampened Tau-related hippocampal CD8^+^ T cell infiltration, decreased neuroinflammation and prevented spatial memory deficits [20]. The intricate interplay between T cell immunity and innate neuroinflammation thus plays an instrumental role in modulating disease progression, with various T cell subsets likely having different impacts.

Regulatory T cells (Tregs) are key modulators of immune responses, playing a critical role in maintaining immunological tolerance and homeostasis, as well as in suppressing excessive immune responses deleterious to the host. We previously showed that early depletion of Tregs accelerates the onset of cognitive deficits in the APPPS1 mouse model of AD-like amyloid pathology, whereas early amplification of Tregs conversely delays the onset of cognitive symptoms [21]. Similar neuroprotective effects of Tregs were subsequently further reported in other mouse models of AD-like pathology [22, 23]. Such beneficial effects were at least partially related to the capacity of Tregs to inhibit detrimental and/or promote beneficial microglia activation profiles and responses [21]. Of note, although early modulation of Tregs did not affect the overall magnitude of microgliosis in APPPS1 mice, it strongly impacted the extent of plaque-associated microglia and translated into altered brain expression of selected potential regulators of microglial function and/or activation [21]. This underlies that refined analysis of glial responses is critically needed for appropriately assessing the impact of Tregs on the modulation of neuroinflammatory profiles. In contrast to microglia, much less is known regarding the impact of Tregs on reactive astrocytes in the context of AD. Whereas peripheral modulation of Tregs did not modify the overall magnitude of cortical and hippocampal astrocytosis in the brain of APPPS1 mice [21], another study reported enhanced global *GFAP* expression and increased astrocytes around amyloid deposits after Tregs amplification in the APPswe/PS1DE9 model [23]. However, the potential impact of Tregs on the phenotype and functional profiles of reactive astrocytes remains unknown.

In this study, we further characterized the impact on reactive astrocytes of Tregs immunomodulation in a mouse model of AD-like amyloid pathology. We found that early depletion of Tregs in APPPS1 mice increases C3-positive reactive astrocytes around large amyloid deposits, consistent with previously observed worsened outcomes in response to Treg depletion. Our data suggest that Tregs contribute to restrain the functional differentiation of pro-inflammatory C3-positive reactive astrocytes associated with advanced amyloid deposits, thus contributing to dampening detrimental neuroinflammation and cognitive deficits.

## Material and methods

### Mice

APPPS1 transgenic mice (Thy1-APP^KM670/671NL^; Thy1- PS1^L166P^) on C57BL/6 background were kindly provided by Prof. Mathias Jucker (Hertie Institute for Clinical Brain Research, University of Tübingen, Tübingen, Germany) and maintained by breeding heterozygotes with wild-type C57BL/6j mice. Animals were kept under strictly monitored specific and opportunistic pathogen-free conditions. All experimental protocols involving animal studies have been approved by the Charles Darwin Ethical Committee for Animal Studies, and were carried out in compliance with European legislation on animal care and scientific experimentation.

### Treg depletion or amplification

Modulation of Tregs was carried out as previously described [21]. Briefly, for Treg depletion, 4- to 5-week-old APPPS1 mice and wild-type littermates were injected intraperitoneally with 200 µg of either purified (BioXCell) or ascite-derived anti-CD25 depleting monoclonal antibody (clone PC61) diluted in phosphate-buffered saline (PBS). Control mice were injected with either PBS or 200 µg of purified control IgG1 (BioXCell). Similar treatments were repeated 4 weeks after the first injection. For amplification of Tregs, 6-week-old APPPS1 mice and wild-type littermates were treated daily for 10 consecutive days with intraperitoneal injections of either PBS or 50,000 IU of recombinant human IL-2 (Proleukin; Novartis) diluted in PBS. Additional daily treatments for 5 days were repeated every 3 weeks. The efficiency of treatments for Treg depletion or amplification was monitored by flow cytometry as previously described [21].

### Immunohistochemistry

At 5 months of age mice were anaesthetized with ketamine (Imalgene®) and xylazine (Rompun™) in 0.9% NaCl solution, then transcardially perfused with ice-cold PBS, followed by a solution of 4% paraformaldehyde in PBS. Brain was harvested, transferred for 48 h at 4 °C in a fresh solution of 4% paraformaldehyde, and then transferred overnight at 4 °C in 30% sucrose/PBS solution. After bisecting along the midline, each hemi-brain was divided into anterior, median and posterior parts by coronal sectioning. Each part was embedded individually in Optimal Cutting Temperature (OCT; WVR) mounting medium, frozen in liquid nitrogen and stored at − 80 °C.

For thin-section analyses, cryosections (8 µm) were prepared from median parts of the brain, using a Leica RM2145 cryostat, and mounted on SuperFrost™ Plus glass slides. Sections were first rinsed in PBS, blocked with PBS + 5% Bovine serum albumin (BSA) + 0.05% Tween 20 + 0.3% Triton X100 for 1 h at room temperature, and then incubated overnight at 4 °C with rabbit anti-Cox2 antibody (1/200) (SP21; Invitrogen) or rat anti-C3 antibody (1/200) (11H9; Novus Biologicals). Following three washes in PBS + 0.5% BSA + 0.05% Tween 20 + 0.3% Triton X100, sections were incubated for 30 min at room temperature with Alexa Fluor 647-conjugated mouse anti-Aβ monoclonal antibody (1/200) (NBP2; Novus Biologicals), Alexa Fluor 488-conjugated mouse anti-GFAP monoclonal antibody (1/500) (GA5; Invitrogen) and either Alexa Fluor 594-conjugated donkey anti-rabbit antibodies or Alexa Fluor 555-conjugated goat anti-rat antibodies (1/500 each) (Invitrogen). After three additional washes brain sections were stained with DAPI (1 µg/ml in PBS) and coverslipped with Immu-Mount™ medium (ThermoShandon).

For 3D modeling analyses, thick cryosections (90 µm) were prepared from median parts of the brain, using a Leica RM2145 cryostat, and transferred in PBS in a 24-well plate for floating-section staining. Sections were first rinsed in PBS, then treated with ice-cold 10% MetOH for 10 min at − 20 °C. Following three washes with PBS, sections were blocked with PBS + 5% BSA + 0.05% Tween 20 for 1 h at room temperature, and then incubated overnight at 4 °C with Alexa Fluor 488-conjugated mouse anti-GFAP monoclonal antibody (1/500) (GA5; Invitrogen) and DyLight 647-conjugated mouse anti-Aβ monoclonal antibody (1/500) (NBP2; Novus Biologicals). Following three final washes in PBS + 0.1% BSA, brain sections were then stained with DAPI (1 µg/ml in PBS), mounted on SuperFrost™ Plus glass slides, and coverslipped with Immu-Mount™ medium (ThermoShandon).

### Epifluorescence microscopy and image analysis

For each mouse, sections were imaged using an Olympus Ix81 microscope equipped with a Retiga Exi camera and an Optigrid II module. Five random images of the cerebral cortex were collected at 20X magnification for each mouse. All sections were imaged using standardized acquisition parameters. Quantifications were carried out by post-processing images with the ImageJ software (http://rsbweb.nih.gov/ij). Percentage of specifically stained area was determined after standardized binarization of fluorescence images. For quantification of astrocyte immunoreactivity in the vicinity of amyloid deposits, we first delineated the proximal surrounding area for each amyloid deposit, which was defined as the limit encompassing twice the radius of the deposit. Amount of GFAP staining within this limit, i.e. corresponding to astrocytes co-localizing with or in close proximity to Aβ deposits, was determined from the binarized images. Pooled data from 4 to 6 mice/group were analyzed, corresponding to *n* = [47–75] deposits of 200–500 µm^2^, *n* = [67–119] deposits of 500–1000 µm^2^ and *n* = [47–64] deposits > 1000 µm^2^ of surface area per group. For the analysis of A1- and A2-like markers, amount of Cox2 or C3 staining within astrocytes, i.e. co-localizing with GFAP staining, was determined from the binarized images. Pooled data from 4–6 mice/group were analyzed, corresponding to *n* = [20–41] deposits of 200–500 µm^2^, *n* = [23–63] deposits of 500–1000 µm^2^ and *n* = [23–33] deposits > 1000 µm^2^ of surface area per group.

### Confocal microscopy and 3D image analysis

For each mouse, sections were imaged using an Olympus FV-1200 inverted confocal microscope equipped with a 488 nm Argon laser and 405 nm, 559 nm and 635 nm diode lasers and gallium arsenide phosphide (GaAsP) detectors for higher sensitivity imaging on the red and far-red rays. Five random images of the cerebral cortex were collected at 40X magnification for each mouse on the full depth of the section with a step of 0.57 µm. All sections were imaged using standardized acquisition parameters. Quantifications were carried out by post-processing images with the Imaris software (Bitplane). For quantification of astrocyte recruitment towards amyloid deposits, we first modeled amyloid deposits in 3D using the “Surfaces” tool of Imaris. We delineated the proximal surrounding volume for each amyloid deposit, which was defined as the limit encompassing twice the radius of the deposit. We then modeled astrocytes’ soma using the “Spots” tool of Imaris, and then used the “Cells” tool to quantify the number of soma, i.e. astrocytes, recruited per plaque. For quantification of astrocytes’ volume, we used the “Surfaces” tool to model as accurately as possible astrocytes’ morphology in 3D. For quantification of astrocytes’ branching complexity, we used the “Filaments” tool to model the ramified architecture of astrocytes according to GFAP staining. We notably performed a Sholl analysis quantifying the number of intersections between astrocyte branches and consecutive concentric spheres originating from astrocytes’ soma and of a radius period of 1 µm. Pooled data from 4–6 mice/group were analyzed, corresponding to *n* = [86–120] deposits of 200–500 µm^2^, *n* = [121–156] deposits of 500–1000 µm^2^ and *n* = [35–70] deposits > 1000 µm^2^ of surface area per group.

### Real-time quantitative PCR

Brains of wild-type and APPPS1 mice previously treated for Tregs depletion or amplification were harvested at 4 months of age after transcardiac perfusion with PBS. Cerebellum was removed and total RNA was extracted from hemi-brains using RNeasy lipid tissue midi kit (Qiagen). Following DNase treatment, RNA quality was verified using an Agilent Bioanalyzer and quantity measured with a Nanodrop 1000 (ThermoFisher Scientific). For cDNA synthesis, 2 µg of total RNA were processed using RT^2^ First Strand kit (Qiagen). Quality control of RNA and cDNA samples was assessed using RT^2^ RNA QC PCR Array (Qiagen). Relative expression of specific mRNAs was assessed by SYBR green-based real-time quantitative PCR using RT^2^ SYBR Green ROX (Qiagen) and LightCycler 96 Instrument (Roche). Specificity of all primers was validated using Primer BLAST database analysis, and PCR efficiency (> 90%) was validated after optimizing the concentration and annealing temperature. Amplification conditions were: 95 °C for 5 min, then 45 cycles at 95 °C for 10 s, 15 s at either 60 °C or 56 °C depending on the primer pairs, and 72 °C for 30 s. A melting curve was generated at the end of amplification cycles for assessing the specificity of the reaction. Hypoxanthine-guanine phosphoribosyltransférase (*HPRT*) and peptidylprolyl isomerase A (*PPIA*) were used as reference housekeeping genes for normalization. Relative expression of genes was evaluated as fold changes using the mean of the control group as reference, and was calculated as 2^−ΔΔCt^. All primers were ordered from Eurogentec (sequences available in Additional file 8: Table S1). Data were analyzed using LightCycler 96 software (Roche) and the R software environment.

### Flow cytometry analysis

Blood samples and splenocytes from anti-CD25- or IL2-treated mice and relevant controls were stained with anti-CD3 FITC (145-2C11, BD Biosciences), anti-CD4 APC-Cy7 (GK 1.5, BD Biosciences), anti-CD8a PerCP-Cy5.5 (53–6.7, BD Biosciences), anti-CD25 APC (PC61, BD Biosciences), anti-CD44 BV421 (IM7, BD Biosciences) and anti-CD62L BV510 (MEL-14, BD Biosciences) antibodies. Cells were then fixed and permeabilized using the Foxp3/Transcription factor fixation/permeabilization Kit (Invitrogen). Cells were then additionally stained with anti-Foxp3 Alexa700 (FJK-16 s, eBioscience), anti-T-bet PE (4B10, BD Biosciences), anti-ROR*γ*T PE-CF594 (Q31-378, BD Biosciences) and anti-GATA3 PE-Cy7 (L50-823, BD Biosciences) antibodies. Alternative staining consisted in anti-CD3 FITC (145-2C11, BD Biosciences), anti-TCRγδ PE-CF594 (GL3, BD Biosciences), anti-CD19 BV421 (1D3, BD Biosciences), anti-NK1.1 Alexa700 (PK136, BD Biosciences), anti-NKp46 PE (REA815, Miltenyi), anti-CD11b PE-Cy7 (M1/70, BD Biosciences), anti-CD11c APC (HL3, BD Biosciences) and anti-Ly6G APC-Cy7 (1A8, BD Biosciences) antibodies. Cells were then fixed using CellFix reagent (BD Biosciences). All samples were processed with a Gallios flow cytometer (Beckman Coulter) and the data analyzed using the Kaluza software (Beckman Coulter).

### Statistical analysis

Data were analyzed using the R software environment (http:// www.R-project.org/). Statistical tests included Mann–Whitney non-parametric test and linear regression models. The specific tests used in each experiment are mentioned in the figure legends. For all statistical analyses, we applied a cut-off value for significance at *p* = 0.05. Non-significant *p*-values < 0.1 were provided for information.

## Results

### Peripheral modulation of Tregs does not alter plaque-associated pan astrocyte reactivity

**Fig. 1:**
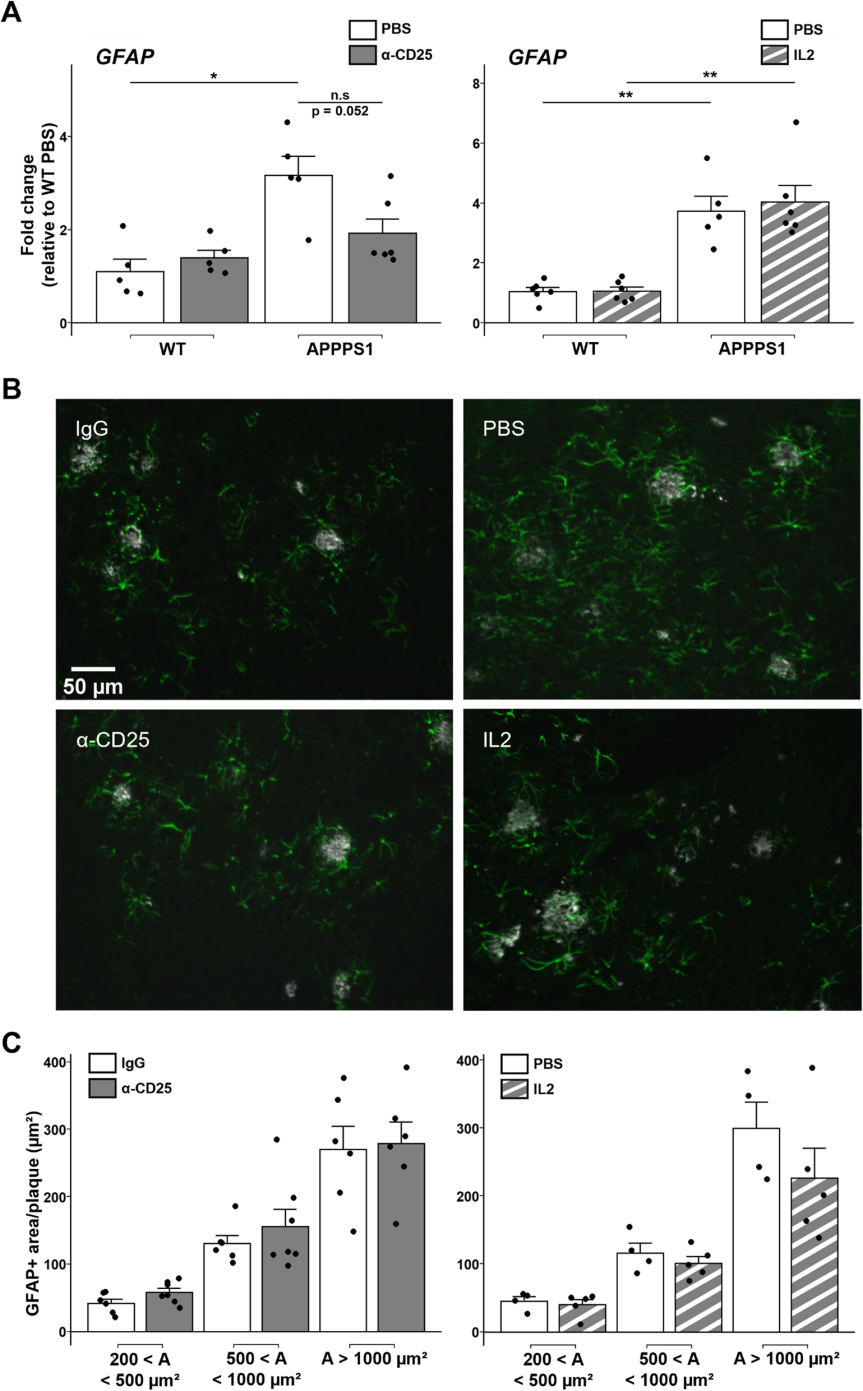
Modulation of Tregs does not alter plaque-associated global astrocyte reactivity. **A** Quantification of mRNA corresponding to the reactive astrocyte marker GFAP in the brain of mice treated with either PBS or anti-CD25 antibody (left), or with either PBS or IL-2 (right). Values were first normalized according to the expression of the housekeeping gene PPIA and then normalized to the mean value of PBS-injected WT control group. **B** Representative images of astrocytes (GFAP, green) associated to amyloid deposits (Aβ, white) in the cerebral cortex of mice treated with either isotypic control antibody (top left) or anti-CD25 (bottom left), or with either PBS (top right) or IL-2 (bottom right). **C** Quantification of GFAP immunoreactivity in close vicinity to amyloid deposits of different size ranges in the cerebral cortex of mice treated with either isotypic control antibody or anti-CD25 (left) or with either PBS or IL-2 (right). Results are represented according to the size of amyloid deposits that astrocytes are recruited towards. Mean +/− SEM (*n* > 20 amyloid deposits from 4–6 mice/ group). Mann–Whitney test: *P < 0.05; **P < 0.01; ***P < 0.001

Our previous work evidenced a beneficial impact of Tregs on disease progression in APPPS1 mice [21]. Whereas early modulation of Tregs did not significantly modulate the overall cortical astrocytosis as measured by standard immunofluorescence analysis, the impact of such Tregs immunomodulation on astrocytes associated with amyloid deposits remains elusive. Here, we refined the analysis of astrocyte reactivity upon Treg modulation. We first quantified the expression of different genes associated with pan astrocyte reactivity, including *GFAP*, asparaginase (*ASPG*), oncostatin M receptor (*OSMR*) and serine protease inhibitor A3N (*SERPINA3N*). Early depletion of Tregs did not significantly affect the expression of *GFAP* in APPPS1 mice (Fig. 1A). Treg depletion did not impact either the expression of other genes associated with pan astrocyte reactivity (Additional file 1: Fig. S1A). Of note, Treg depletion was associated with significantly increased cerebral expression of the pan-astrocytic marker *ALDH1L1* in WT mice, suggesting imbalanced homeostasis of astrocytes at steady state upon Treg depletion (Additional file 1: Fig. S1C). Selective amplification of Tregs via low-dose IL-2 treatment did not significantly modulate the expression of *GFAP* in APPPS1 mice (Fig. 1A). Similarly, selective amplification of Tregs did not impact the expression of any genes associated with pan astrocyte reactivity or *ALDH1L1* (Additional file 1: Fig. S1B-C).

We then further investigated the extent of astrocytosis in the close vicinity of amyloid deposits, upon either amplification or depletion of Tregs. We thus quantified the immunoreactive area for GFAP, according to three size ranges of cortical amyloid deposits. The extent of plaque-associated GFAP was not affected for any size range of amyloid deposits in the brain of Treg-depleted APPPS1 mice as compared to non-depleted littermates (Fig. 1B-C). Astrocyte reactivity in the vicinity of amyloid deposits was also not significantly modified upon Treg amplification in APPPS1 mice (Fig. 1B-C). These results suggest that peripheral modulation of Tregs does not significantly alter the magnitude of global cerebral astrocytosis, nor modulate global astrocyte reactivity in the close vicinity of cortical amyloid deposits.

### Modulation of Tregs does not affect the number nor the volume of plaque-associated reactive astrocytes

**Fig. 2:**
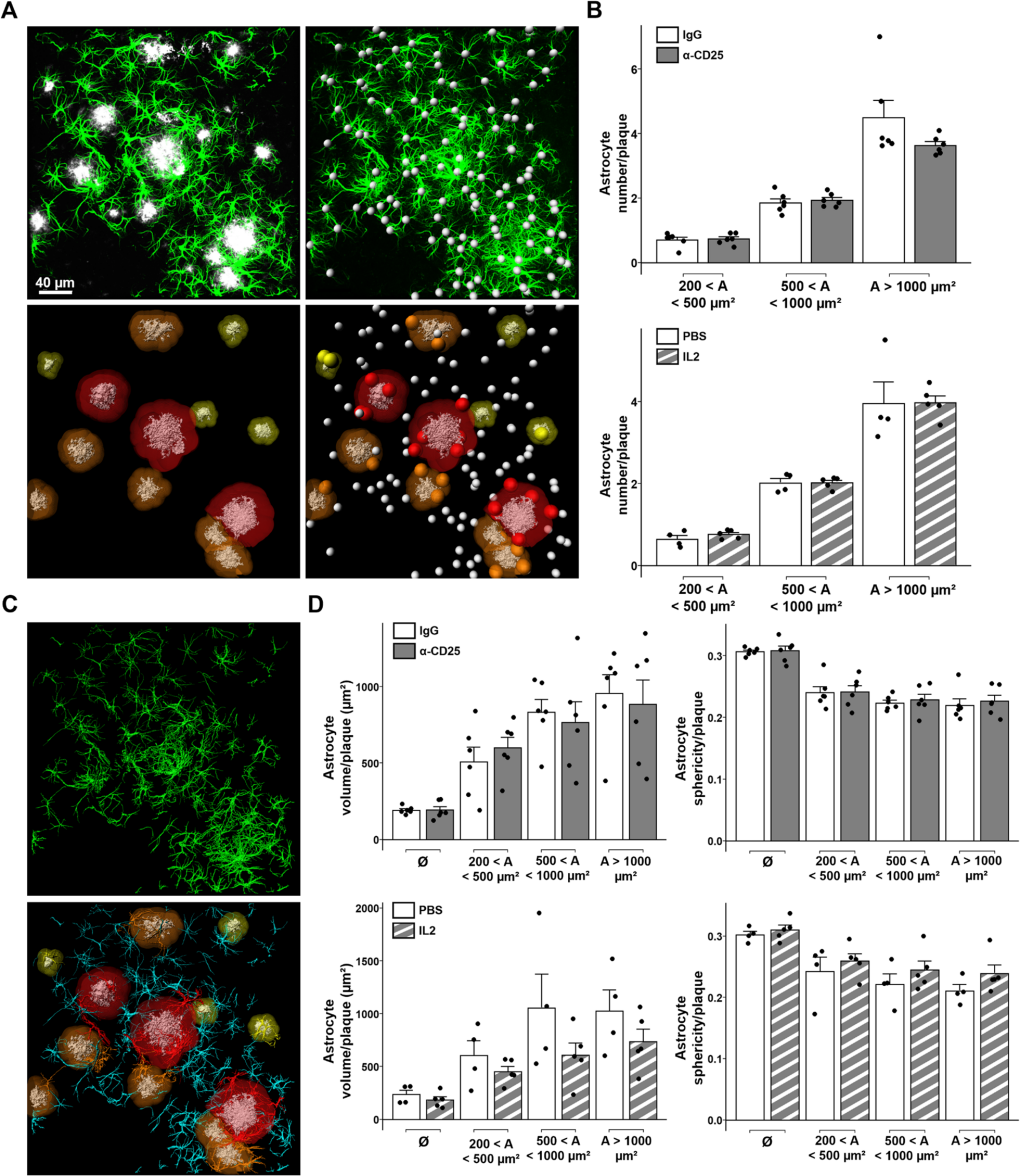
Modulation of Tregs does not affect the volume and sphericity of plaque-associated astrocytes. **A** Illustration of the 3D modeling process for analyzing astrocytes’ recruitment around amyloid deposits. Top left: representative raw image of astrocytes (GFAP, green) associated to cortical amyloid deposits (Aβ, white) from a 90-µm-thick cortical section. Top right: modeling of astrocytes’ soma (white). Bottom left: 3D-modeling of amyloid deposits (white) and their proximal environment (yellow to red, according to size range). Bottom right: representative image of astrocytes’ soma recruitment in the vicinity of amyloid deposits. Recruited astrocytes’ soma are represented as colored spheres according to the size of their corresponding amyloid deposit, unrecruited astrocytes’ soma are represented as white spheres. **B** Quantification of astrocyte recruitment in close vicinity to amyloid deposits of different size ranges in the cerebral cortex of APPPS1 mice treated for either the depletion (top) or amplification of Tregs (bottom). **C** Illustration of the 3D modeling process for analyzing astrocytes’ volume. Top: 3D modeling of astrocytes. Bottom: representative image of modeled astrocytes and amyloid plaques. Astrocytes in the vicinity of amyloid deposits are colored according to the size of their corresponding amyloid plaque, unrecruited astrocytes are represented in cyan. **D** Quantification of astrocytes’ volume (left) or sphericity (right) according to their proximity to amyloid deposits of different size ranges in the cerebral cortex of APPPS1 mice treated for either the depletion (top) or amplification of Tregs (bottom). Results are represented according to the size of amyloid deposits. Unrecruited astrocytes are referenced as Ø. Mean +/− SEM (*n* > 30 amyloid deposits from 4–6 mice/group). Mann–Whitney test: *P < 0.05; **P < 0.01; ***P < 0.001

To further investigate the potential impact of Tregs on plaque-associated astrocytosis, we quantified the actual number of reactive astrocytes in the vicinity of amyloid deposits. 90-µm-thick floating sections were analyzed by confocal imaging across their full depth, and subsequently reconstructed in 3D using Imaris software. Amyloid plaques and their proximal volume were modeled, as well as astrocytes’ soma. Quantification of astrocytes’ soma in the vicinity of amyloid plaques evidenced no significant impact of either Treg depletion or amplification on the number of recruited reactive astrocytes compared to their respective controls (Fig. 2A-B).

We then evaluated the volumetric morphology of astrocytes in the vicinity of amyloid deposits. We modeled astrocytes in 3D and classified them according to their proximity to amyloid deposits as previously described (Fig. 2C). Whereas the mean volume of plaque-associated astrocytes was higher than the volume of non-recruited astrocytes, no significant difference in their volume was observed upon either amplification or depletion of Tregs, in any plaque size-category (Fig. 2D). Finally, we evaluated the sphericity of plaque-associated astrocytes, as a complementary morphological feature that could reflect variations in reactive astrocytes. Again, no significant difference was observed upon Treg modulation (Fig. 2D). Hence, the number and basic morphological features of astrocytes recruited towards amyloid deposits do not seem to vary according to the modulation of Tregs.

### Modulation of Tregs does not affect the branching complexity of astrocytes

**Fig. 3:**
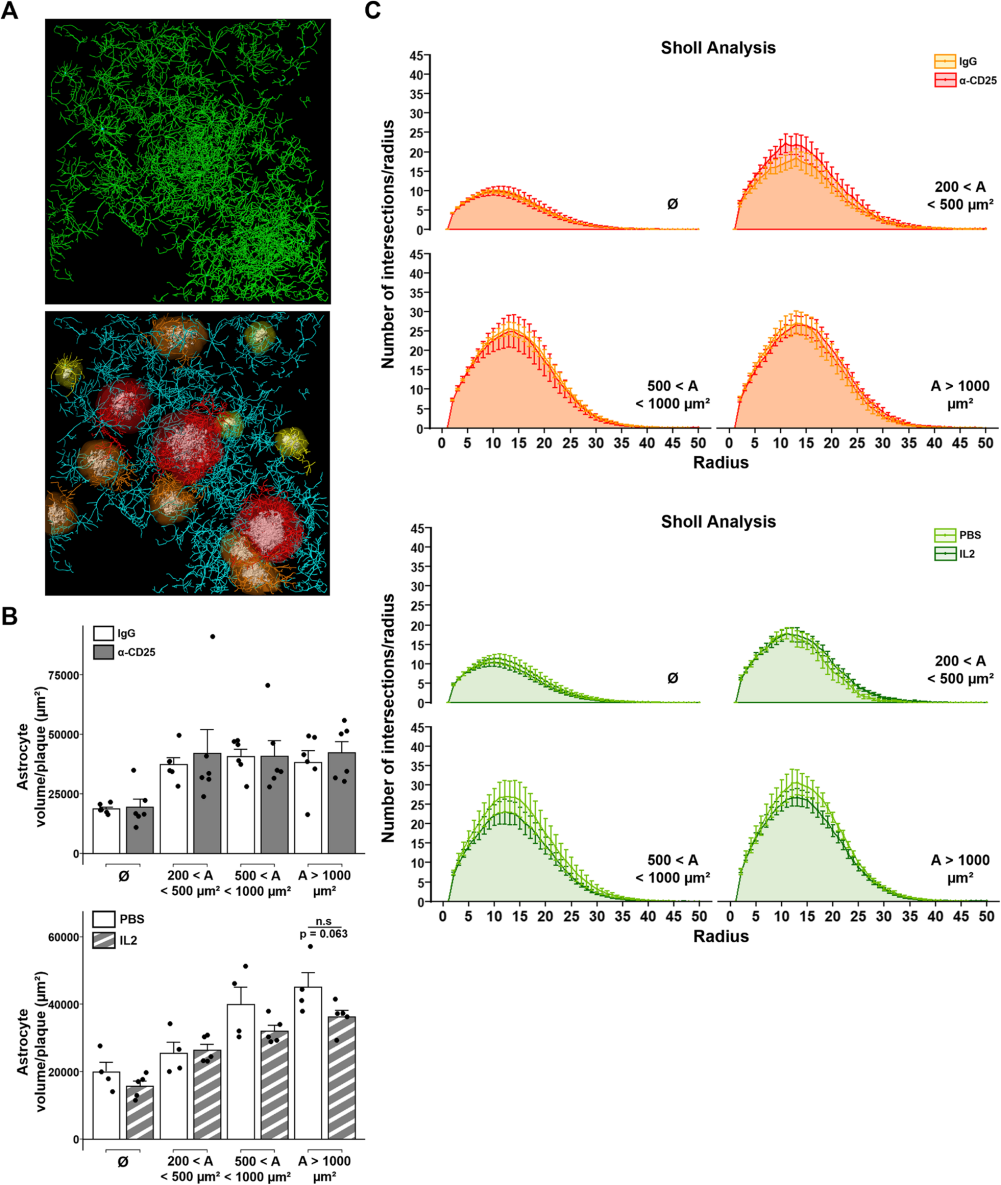
Modulation of Tregs does not impact the morphology and branching complexity of plaque-associated astrocytes. **A** Illustration of the 3D modeling process for analyzing astrocytes’ branching complexity around amyloid deposits. Top panel: 3D modeling of astrocytes branching hierarchy. Bottom panel: representative image of modeled astrocytes and amyloid plaques. Astrocytes in the vicinity of amyloid deposits are colored according to the size of their corresponding amyloid plaque, unrecruited astrocytes are represented in cyan. **B** Quantification of astrocytes’ bounding box volume in close vicinity to amyloid deposits of different size ranges in the cerebral cortex of APPPS1 mice treated for either the depletion (top) or amplification of Tregs (bottom). **C** Sholl analysis of the number of intersections between astrocyte branches and consecutive concentric spheres centered on astrocytes’ soma and of a radius period of 1 µm, of APPPS1 mice treated for either the depletion of Tregs (top) or the amplification of Tregs (bottom). Results are represented according to the size of amyloid deposits astrocytes are recruited towards. Unrecruited astrocytes are referenced as Ø. Mean +/− SEM (*n* > 30 amyloid deposits from 4–6 mice/ group). Mann–Whitney test (**A**-**B**): *P < 0.05; **P < 0.01; ***P < 0.001. Linear regression (**C**): *P < 0.05; **P < 0.01; ***P < 0.001

We then assessed more complex morphological features of astrocytes, such as the branching complexity, by reconstructing the ramified architecture of astrocytes according to GFAP staining (Fig. 3A). We first evaluated the minimal volume of the box encompassing all processes of each individual astrocyte, which reflects the total volume of occupancy and interaction capacity of astrocytes’ ramifications. We did not find any statistical difference in the total volume occupied by astrocytes processes in the cortex of either Treg-amplified or -depleted APPPS1 mice (Fig. 3B).

To further characterize the branching complexity of astrocytes we evaluated a large variety of morphological parameters, including (i) the cumulated length of processes, (ii) the branching depth index, which reflects the maximum branching complexity, (iii) the total number of branching points, and (iv) the total number of terminal points. No differences were found for any of these parameters between plaque-associated astrocytes in treated and untreated APPPS1 mice, upon either amplification or depletion of Tregs (Additional file 2: Fig. S2A-B). Finally, we evaluated the spatial repartition of astrocytic branching complexity via a Sholl analysis, which assesses the number of intersections between astrocytes’ processes and concentric spheres originating from the soma center. We first evaluated the density of processes in each concentric sphere by quantifying the area under the curve obtained with the Sholl analysis. No difference between groups was identified with this approach (Additional file 2: Fig. S2C). We then used linear regression models for further evaluating potential differences between groups, and no statistical correlation between the Sholl profiles and Treg-modulating treatments was identified (Fig. 3C). Overall, these results do not evidence any significant difference in astrocytic morphology, branching complexity and recruitment towards amyloid deposits in the cortex of either Treg-depleted or Treg-amplified APPPS1 mice compared to Treg-unmodulated animals.

### Early depletion of Tregs increases plaque-associated C3^+^ astrocytes

**Fig. 4:**
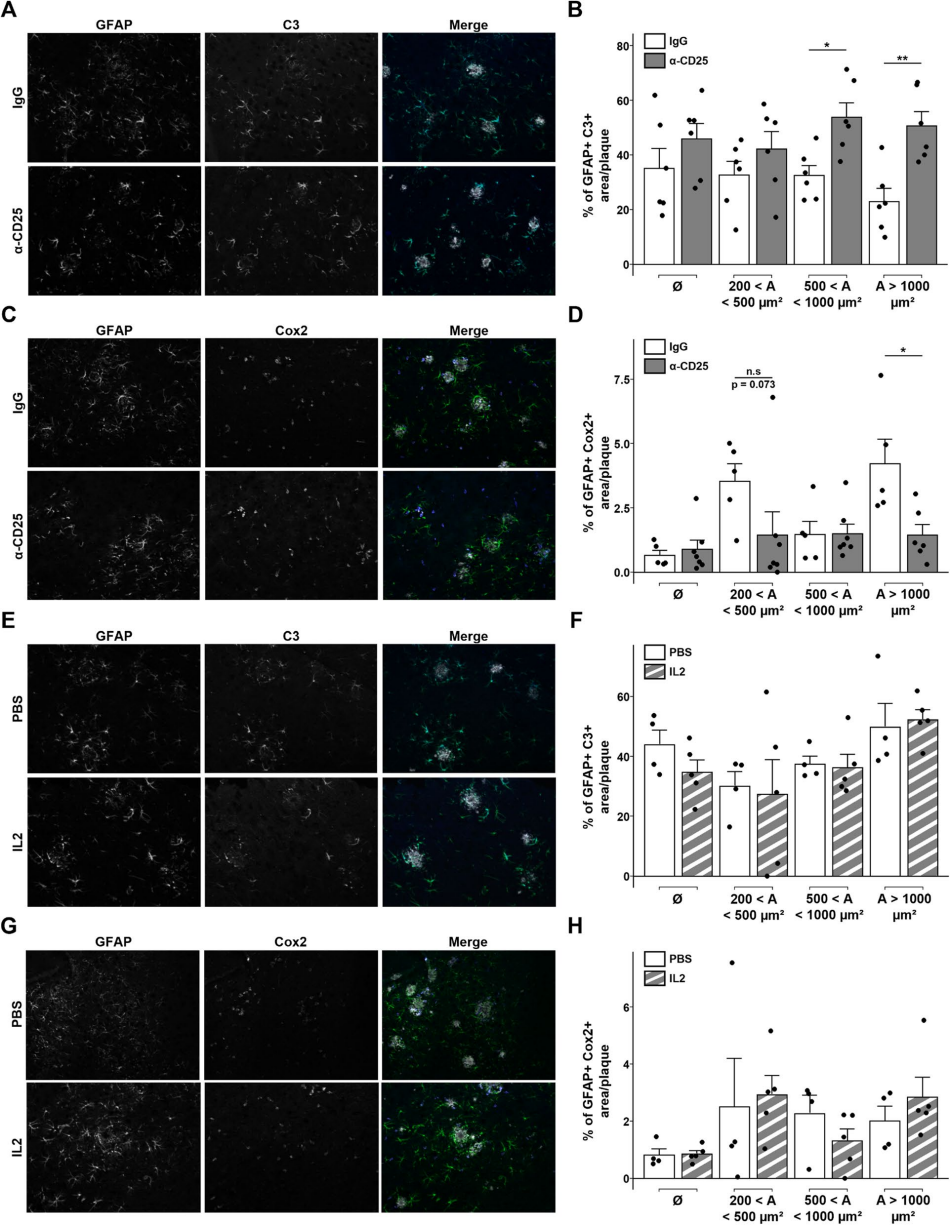
Early depletion of Tregs modulates the phenotype of plaque-associated reactive astrocytes. **A**,**E** Representative images of astrocytes (GFAP, green) and their expression of the A1-like astrocytic marker C3 (blue) associated to amyloid deposits (Aβ, white) in the cerebral cortex of mice treated with either a control or anti-CD25 antibody (**A**) or with either PBS or IL-2 (**E**). **B**, **F** Quantification of GFAP area colocalized with C3 in close vicinity to amyloid deposits of different size ranges in the cerebral cortex of mice treated with either a control or anti-CD25 antibody (B) or with either PBS or IL-2 (**F**). **C**,**G** Representative images of astrocytes (GFAP, green) and their expression of the A2-like astrocytic marker Cox2 (blue) associated to amyloid deposits (Aβ, white) in the cerebral cortex of mice treated with either a control or anti-CD25 antibody (**C**) or with either PBS or IL-2 (**G**). **D**,**H** Quantification of GFAP area colocalized with Cox2 in close vicinity to amyloid deposits of different size ranges in the cerebral cortex of mice treated with either a control or anti-CD25 antibody (**D**) or with either PBS or IL-2 (**H**). Results are represented according to the size of amyloid deposits. Unrecruited astrocytes are referenced as Ø. Mean +/− SEM (*n* > 10 amyloid deposits from 4–6 mice/ group). Mann–Whitney test: *P < 0.05; **P < 0.01; ***P < 0.001

To further investigate whether the modulation of Tregs may impact the functional profiles of reactive astrocytes, we analyzed by immunofluorescence the expression of markers for neurotoxic A1-like and neuroprotective A2-like reactive astrocyte subsets. Interestingly, early depletion of Tregs significantly increased the extent of C3 expression, an A1-like astrocyte marker, in reactive astrocytes located in the vicinity of amyloid deposits displaying a surface area superior to 500 µm^2^, compared to control-treated animals (Fig. 4A-B). Of note, no significant difference in C3 expression was observed in non-recruited astrocytes. Conversely, the expression of the A2-like astrocytic marker cyclo-oxygenase 2 (Cox2) was significantly decreased in astrocytes associated with larger amyloid deposits of a surface area superior to 1000 µm^2^ in Treg-depleted APPPS1 mice, compared to control-treated animals (Fig. 4C-D). Similar to C3, no difference in Cox2 expression was observed in non-recruited astrocytes upon Treg depletion.

These results support that Treg depletion enhances the polarization towards a C3^+^ phenotype of reactive astrocytes associated with medium to large amyloid deposits. Treg-amplified APPPS1 mice did not display any modification in the expression of the A1-like astrocytic marker C3 when compared to control PBS-treated APPPS1 mice, either in non-recruited or plaque-associated astrocytes (Fig. 4E-F). Similar analysis for the A2-like marker Cox2 showed that Treg-amplified APPPS1 mice once again did not show altered levels of Cox2 expression in astrocytes, whether recruited or not to cortical amyloid deposits, when compared to control PBS-treated APPPS1 mice (Fig. 4G-H). Treg amplification thus does not seem to significantly impact the functional profile of plaque-associated astrocytes in APPPS1 mice. Overall, our data suggest that Tregs restrain the functional differentiation of plaque-associated reactive astrocytes into potentially pro-inflammatory C3^+^ A1-like neurotoxic subsets in response to amyloid pathology, thus dampening detrimental neuroinflammation in AD-like pathology.

### Early modulation of Tregs affects the expression of some A1- and A2-like markers of astrocyte reactivity in healthy and APPPS1 mice

**Fig. 5:**
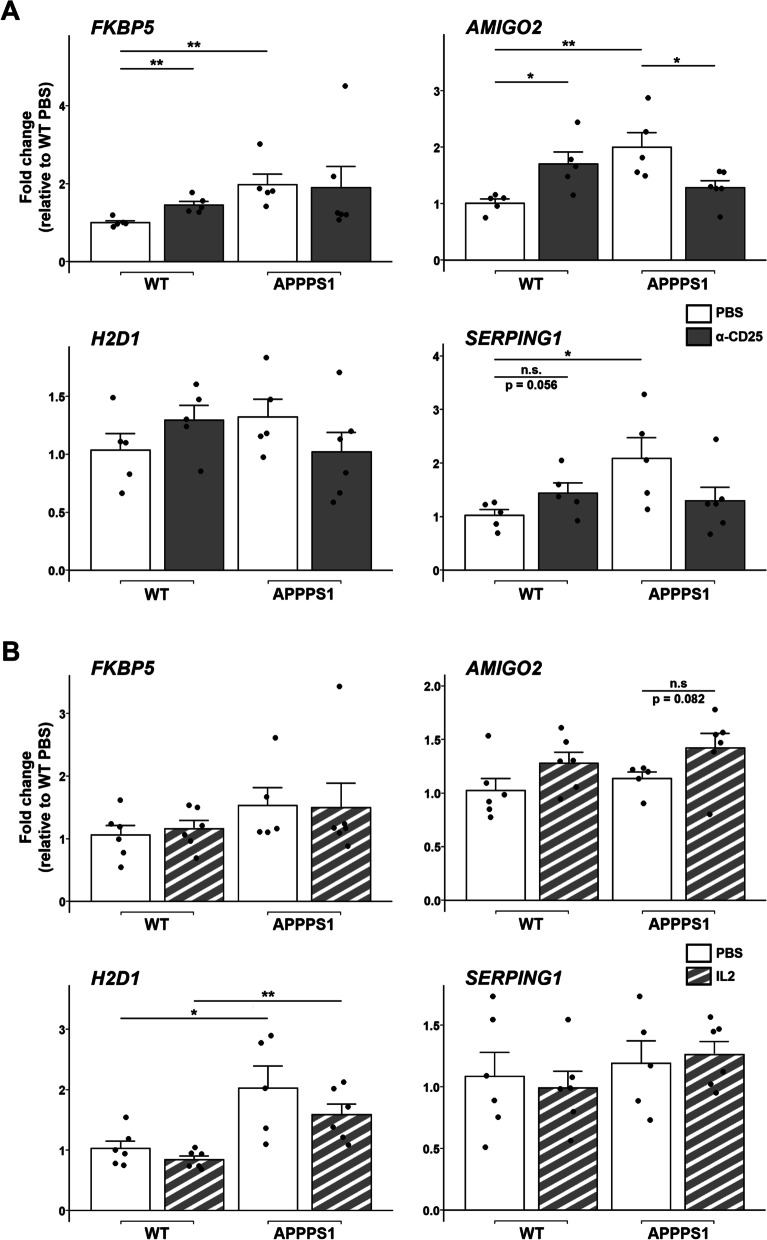
Depletion of Tregs affects the expression of several A1-like astrocytic markers. **A**, **B** Quantification of mRNA coding for the A1-like reactive astrocyte markers FKBP5, AMIGO2, H2D1 and SERPING1 in the brain of mice treated with either PBS or anti-CD25 antibody (**A**), or with either PBS or IL-2 (**B**). Values were first normalized according to the expression of the housekeeping gene PPIA and then normalized to the mean value of PBS-injected WT control group. Mean +/− SEM (from *n* = 5–6 mice/ group). Mann–Whitney test: *P < 0.05; **P < 0.01; ***P < 0.001

To further assess the impact of Treg modulation on the functional profiles of reactive astrocytes, we evaluated the expression of additional markers of reactive astrocyte subsets in the brain of Treg-modulated APPPS1 mice. Neither depletion nor amplification of Tregs significantly impacted the expression of several other A1-like (*FKBP5*, *H2D1, SERPING1*) or A2-like astrocytic markers (*S100A10, EMP1, TM4SF1)* in APPPS1 mice (Fig. 5A-B and Additional file 3: Fig. S3A-B). However, the modulation of Tregs affected the expression of the A1-like astrocytic marker *AMIGO2*, which decreased in Treg-depleted APPPS1 mice (Fig. 5A). We then investigated the expression of C1qa, TNFα, and IL-1α, three factors released by activated microglia and involved in the functional differentiation of reactive astrocytes into A1-like subsets [10]. Neither depletion nor amplification of Treg significantly impacted the expression of IL-1α, TNFα or C1qa in APPPS1 mice. (Additional file 4: Fig. S4A-B). Overall, these data suggest that in pathological conditions Tregs might contribute to regulate the balance of functional astrocytes subsets by more complex mechanisms than merely modulating the expression of polarizing factors promoting A1-like reactive astrocytes. Additionally, our data suggest a lack of consistency in the expression pattern across several supposedly A1-like markers.

We further evaluated the expression of these markers in the brain of Treg-modulated healthy mice. Interestingly, early Treg depletion significantly increased the expression of two A1-like markers, *FKBP5* and *AMIGO2*, in healthy animals (Fig. 5A). Of note, early Treg depletion also significantly increased the expression of the A2-like marker *TM4SF1* in such animals (Additional file 3: Fig. S3A). Conversely, early amplification of Tregs significantly decreased the expression of C1qa in the brain of WT mice (Additional file 4: Fig. S4B). Neither depletion nor amplification of Tregs significantly impacted the expression of either IL-1α or TNFα in WT mice. (Additional file 4: Fig. S4A-B). Furthermore, early amplification of Tregs decreased the expression of the A2-like astrocyte marker *S100A10* in healthy animals (Additional file 3: Fig. S3B).

Altogether, these data suggest that Tregs may contribute to regulate the balance and/or homeostasis of functional astrocytes subsets even at steady state in the healthy brain, potentially by down-modulating the expression of C1qa. Moreover, the counter-intuitive variation in A1- and A2-like markers, as well as related promoting factors, upon Treg-modulation further questions their relevance as selective markers of given astrocyte subsets.

### Modulation of Tregs does not consistently affect the frequency of other immune populations

To better decipher if other immune populations might be involved in the observed changes in astrocytes states, we assessed in further details the impact of each type of immunomodulatory treatment on multiple other immune populations. We previously reported that Tregs modulation via anti-CD25 or chronic low-dose IL-2 treatment selectively impacted Tregs without significantly affecting CD25^+^ conventional CD4^+^ T cells in APPPS1 mice [21]. Here, we further investigated whether depletion of Tregs impacted the peripheral frequencies of B cells, monocytes, dendritic cells, NK cells, NK T cells, γδ T cells, as well as other functional T cell subsets such as Th1, Th17, Th2 or CD25^+^CD8^+^ T cells. Upon Treg depletion we did not observe significant nor consistent variations in the frequency of any other immune subset, except a significant decrease in a minor CD25^+^Foxp3^+^CD8^+^ T cell population, exclusively in the spleen (Additional file 5: Fig. S5). Similarly, we did not observe any consistent variation of any other immune populations after amplification of Tregs, except a significant increase in CD25^+^Foxp3^−^CD8^+^ T cells, exclusively in the blood of APPPS1 mice (Additional file 6: Fig. S6). Finally, we investigated if immunomodulatory treatments could selectively affect functional subsets of Tregs, i.e. naive *vs* antigen-experienced Tregs.

Relative peripheral frequencies of naive (CD62L^+^CD44^−^) and activated/memory (CD62L^−^CD44^+^) CD4^+^Foxp3^+^ Tregs did not differ between WT and APPPS1 mice, nor were they consistently altered by either anti-CD25 or low-dose IL-2 treatment (Additional file 7: Fig. S7). Altogether, these results suggest that neither depletion nor amplification of Tregs significantly nor consistently impact other peripheral immune populations in our settings, and does not consistently impact the ratio between naive and antigen-experienced Tregs.

## Discussion

Our study suggests that Tregs play a role in the regulation of astrocyte functionality and reactivity in a mouse model of AD-like amyloid pathology. Whereas modulation of Tregs did not significantly impact pan reactivity and morphological features of astrocytes, transient depletion of Tregs upregulated the expression of the A1-like reactive marker C3 in astrocytes while simultaneously down-regulating the expression of the A2-like reactive marker Cox2 in astrocytes around large amyloid deposits. Rather than regulating the overall magnitude of astrocytosis, Tregs thus appear to modulate and/or fine-tune the polarization of reactive astrocytes in the brain of APPPS1 mice.

Reactive astrocytes have been shown to be critical regulators of neuronal function and synapse homeostasis in neurodegenerative diseases including AD. C3^+^ A1-like reactive astrocytes appear to be neurotoxic, naturally occurring in aging and seemingly involved in the neuroinflammation associated with neurodegeneration. A recent report suggested that a substantial component of the neurotoxic activity of reactive astrocytes was exerted through the release and delivery of saturated lipids [24]. To our knowledge, whether this mechanism is involved in the pathophysiology of AD still remains unknown. Besides, deficiency in C3 seems to protect against cognitive decline in a mouse model of AD-like amyloid pathology, suggesting C3 as another factor secreted by A1-like astrocytes that could potentially contribute to their neurotoxicity [13]. Furthermore, inactivation of C3aR, the receptor to C3, in a mouse model of AD-like Tau pathology improved cognitive functions and altered activation of astrocytes [25]. Activation of the complement pathway thus seems to be closely associated with the progression of neurodegeneration in AD. Direct implication of astrocytes in this process has been suggested as activation of astrocytes, notably by aggregated Aβ, drives the production of C3 in an NF-κB-dependent manner [26].

Our previous studies demonstrated a beneficial role of Tregs in the APPPS1 mouse model of AD-like amyloid pathology [21]. The transient depletion of Tregs accelerated the onset of cognitive deficits, accompanied by a decrease in microglia associated with amyloid deposits. Conversely, amplification of Tregs delayed the onset of cognitive deficits and upregulated plaque-associated microglia in APPPS1 mice. We thus previously suggested that the beneficial impact of Tregs could be mediated at least in part by modulating microglial responses. Activated microglial cells can indeed mediate a large variety of effector functions, including the production of cytokines and pro-inflammatory factors. Among such secreted factors, TNFα, IL-1α and C1q are preferentially produced by activated microglia and are deemed to be responsible for the polarization of A1-like reactive astrocytes [10]. Release of C1q by microglial cells has been directly linked with synaptic pruning and elimination during brain development [27]. An increase in C1q secretion in the brain of AD-like mouse models has also been linked with neurotoxicity and synapse loss associated with amyloid deposition [28]. Our data now support that transient Treg depletion promotes A1-like phenotypes in reactive astrocytes, as characterized by C3 immunoreactivity, in the vicinity of large cortical amyloid deposits, while down-regulating A2-like astrocytic signatures in plaque-associated astrocytes. Of note, while our study suggests that Tregs alter the balance of reactive astrocyte subsets based on individual analysis of GFAP^+^/Cox2^+^ and GFAP^+^/C3^+^ cells, complementary studies are still needed to fully decipher if these phenotypic markers are strictly mutually exclusive or could potentially be co-expressed in given astrocyte subsets. Our data further suggest that modulation of Tregs does not significantly nor consistently alter the expression of microglial factors known to promote the polarization of astrocytes into A1-like phenotypes, supporting that Tregs might contribute to regulate the balance of functional astrocytes subsets by more complex mechanisms. Here, we did not observe any significant impact on other peripheral immune populations than Tregs, after either type of immunomodulatory treatment, supporting that the impact on astrocytes reactivity is indeed linked to the modulation of Tregs themselves. Previous studies reported that Treg modulation altered the ratio of CD4^+^ and CD8^+^ T cells in another mouse model of AD-like pathology [22]. Although we did not identify any consistent variation in the peripheral frequencies of multiple other immune populations, Tregs may still impact neuroinflammation via modulating the functionality of selected immune cells, or by modulating their recruitment through the choroid plexus, as a critical gate of entry for leukocytes towards the central nervous system [29]. While extremely few parenchymal CD3^+^Foxp3^+^ Tregs can be observed in our model before or after Treg modulation (data not shown), it remains to be defined whether and how Tregs might modulate disease-related neuroimmune processes at brain borders. Particularly, the impact of Tregs on the meningeal immune compartment, known to play key roles in neuroimmune interactions, remains to be further defined. Deeper characterization of immune cells in the periphery, parenchyma and at the borders of the central nervous system, upon modulation of Tregs in AD-like mice, would allow to better understand their mechanistic involvement in modulating microglial and astrocytic responses along disease progression.

Increasing evidence report the importance of A1-like astrocytes in neurodegenerative and neuroinflammatory processes [10, 30, 31]. Nevertheless, the classification of reactive astrocytes into either neurotoxic A1 or neuroprotective A2 phenotypes now clearly appears oversimplified. In this line, recent studies evidenced a more complex heterogeneity of reactive astrocytes in both humans and mice [32, 33]. Our data yielded conflicting results regarding the variation in the expression of supposedly A1-like and A2-like genes in response to amyloid pathology, as well as upon Treg modulation. Markers of both A1-like and A2-like reactive astrocytes were upregulated in APPPS1 as compared to WT mice (Fig. 5 and Additional file 3: Fig. S3). In addition, Treg depletion appeared to upregulate the expression of several A1-like astrocytic markers (e.g. *FKBP5*, *AMIGO2, SERPING1*) in healthy animals, while also upregulating the expression of *TM4SF1*, an A2-like astrocyte marker. Similarly, co-existing A1-like C3^+^ and A2-like S100A10^+^ astrocytes have been previously reported in post-mortem brains of patients with AD [11]. Furthermore, the recent characterization of reactive astrocytes in mouse models of Tau or amyloid pathologies does not match the A1/A2 paradigm [34]. Multiple subtypes of reactive astrocytes may thus occur concomitantly and play an instrumental role in the pathophysiology of AD, as supported by recent single-nuclei RNAseq studies [33]. Furthermore, transcriptional regulators such as SOCS3 in astrocytes have been shown to down-regulate simultaneously the expression of genes associated with both A1-like and A2-like reactive phenotypes [35]. Several of the described A1-like and A2-like markers appear to be modulated in response to this signaling pathway. This suggests a potentially more complex spectrum of astrocyte reactivity than the bipolar view recently proposed. Characterization of the disease-associated astrocytes (DAAs), a population of reactive astrocytes, further highlights the need for better understanding and characterizing the complex variety of reactive astrocytes [36]. Whereas DAAs have been described as sharing some markers with A1-like astrocytes, other recently characterized reactive astrocyte clusters enriched in the inflamed brain do not appear to overlap with the profiles of DAAs [32]. This discrepancy in astrocyte subsets associated with neuroinflammatory conditions strengthens the need to refine the characterization and nomenclature of reactive astrocytes phenotypes. Notably, efforts are now being made to better integrate the current knowledge on reactive astrocytes and their heterogeneity, as recently highlighted in a consensus statement [37]. In this line, further characterization by transcriptomic analyses and functional validation of the modulation of astrocyte reactivity by peripheral Tregs appear both of high importance to decipher the interplay between astrocyte reactivity and the pathophysiology of AD. Although the currently described markers of functional subsets are of interest for the study of reactive astrocytes, ongoing research in the field should yield refined tools and markers of interest in the near future, which are critically needed for better deciphering the complexity of astrocytes reactivity. Of note, efforts to better characterize reactive astrocytes evidenced modifications of their transcriptomic profiles depending on the inflammatory status but also their spatial location in the brain [32]. This suggests a complex and intricate relation between the topography of brain inflammation and the response of reactive astrocytes in pathological contexts.

Interestingly, although we identified variations in astrocyte phenotypes upon Treg immunomodulation, we did not observe any significant difference in astrocyte number, morphology or branching hierarchy. Whereas the study of astrocyte morphology is considered a potent tool to evaluate astrocyte reactivity [7], the use of GFAP immunohistochemistry for that purpose could be debated, as it might not reflect the full complexity of astrocyte finer morphological changes [38, 39]. In this line, our study suggests that fine phenotypic variations of reactive astrocytes may remain unnoticeable through a classical although detailed evaluation of astrocyte morphology. This might be related to the already high neuroinflammatory status found in AD and related mice models. The need for more selective and validated markers of reactive astrocyte phenotypes seems particularly critical in such a neuroinflammatory context associated with neurodegeneration.

In conclusion, Tregs appear to modulate astrocyte reactivity in AD-like amyloid pathology, by dampening C3^+^ A1-like phenotypes in favor of Cox2^+^ A2-like subsets of reactive astrocytes. This expected beneficial effect of Tregs on astrocytes functional polarization in the context of AD-like pathology might partly relate to their apparent intrinsic capacity at modulating astrocytes homeostasis and restraining the development of A1-like astrocytes in steady state healthy conditions. In contrast to Treg depletion, the amplification of Tregs by chronic low-dose IL-2 treatment did not significantly modulate astrocyte functionality in our study. Hence, Tregs may play a key role in restraining overt and/or dysregulated reactivity of pro-inflammatory astrocytes, in both healthy conditions and in response to amyloid pathology, with a threshold effect that might not be further enhanced upon Tregs amplification. Whether Tregs can also modulate astrocyte activity in a more subtle way remains to be further investigated. Future studies will be needed to further decipher the mechanistic bases of the functional modulation of astrocytes by Tregs, which remains critical for better understanding the complex role of the peripheral–central immune crosstalk in the pathophysiology of AD. By modulating both activated microglia and reactive astrocytes, Tregs appear as key modulators of neuroinflammatory processes in AD-like pathology. Treg-targeting peripheral immunomodulatory approaches thus represent promising innovative immunotherapeutic strategies for rebalancing detrimental neuroinflammatory responses in AD and potentially other neurodegenerative disorders.

## Supplementary Information

**Additional file 1: Figure S1.** Modulation of Tregs does not affect the expression of astrocyte pan-reactivity markers. **A-B.** Quantification of mRNA corresponding from left to right to the genes coding for the pan-reactive astrocyte markers ASPG, OSMR and SERPINA3N in the brain of mice treated with either PBS or anti-CD25 antibody (A), or with either PBS or IL-2 (B). **C.** Quantification of mRNA corresponding to the constitutive astrocyte marker ALDH1L1 in the brain of mice treated with either PBS or anti-CD25 antibody (left), or with either PBS or IL-2 (right). Values were first normalized according to the expression of the housekeeping gene PPIA and then normalized to the mean value of PBS-injected WT control group. Mean +/− SEM (from 5–6 mice/ group). Mann–Whitney test: *P < 0.05; **P < 0.01; ***P < 0.001.

**Additional file 2: Figure S2.** Modulation of Tregs does not affect the morphology and branching complexity of plaque-associated astrocytes. **A-B.** Analysis of astrocytes branching complexity in the cerebral cortex of APPPS1 mice treated for either the depletion (A) or amplification of Tregs (B). Quantification of i) astrocytes processes cumulated length (top-left), ii) astrocytes branching depth index (top-right), iii) astrocytes number of processes branching points (bottom-left) and iv) astrocytes number of processes terminal points (bottom-right) in close vicinity to amyloid deposits of different size ranges are shown for each treatment. **C.** Mean area under curve of the Sholl analysis presented in Fig. 3C on APPPS1 mice treated for either the depletion or the amplification of Tregs. Results are represented according to the size of amyloid deposits. Unrecruited astrocytes are referenced as Ø. Mean +/− SEM (*n* > 30 amyloid deposits from 4–6 mice/ group). Mann–Whitney test: *P < 0.05; **P < 0.01; ***P < 0.001.

**Additional file 3: Figure S3.** Modulation of Tregs does not consistently affect the gene expression of several A2-like astrocytic markers. **A-B.** Quantification of mRNA coding for the A2-like astrocytic markers S100A10, EMP1 and TM4SF1 in the brain of mice treated with either PBS or anti-CD25 antibody (A) or with either PBS or IL-2 (B). Values were first normalized according to the expression of the housekeeping gene PPIA and then normalized to the mean value of the PBS-injected WT control group. Mean +/− SEM (from *n* = 5–6 mice/ group). Mann–Whitney test: *P < 0.05; **P < 0.01; ***P < 0.001.

**Additional file 4: Figure S4.** Modulation of Tregs does not consistently affect the expression of several A1-like reactive astrocyte polarization factors. **A-B.** Quantification of mRNA coding for the A1-like reactive astrocyte polarization factors C1qa, TNFα, and IL-1α in the brain of mice treated with either PBS or anti-CD25 antibody (A) or with either PBS or IL-2 (B). Values were first normalized according to the expression of the housekeeping gene PPIA and then normalized to the mean value of the PBS-injected WT control group. Mean +/− SEM (from *n* = 5–6 mice/ group). Mann–Whitney test: *P < 0.05; **P < 0.01; ***P < 0.001.

**Additional file 5: Figure S5.** Depletion of Tregs does not consistently affect other peripheral immune populations. Percentage of B cells (CD19^+^) and T cells (CD3^+^) (A), CD4^+^ and CD8^+^ T cells within CD3^+^ cells (B), Th1 (T-bet^+^), Th17 (RORγT^+^) and Th2 (GATA3^+^) within CD4^+^ T cells (C), CD25^+^Foxp3^+^ and CD25^+^Foxp3^−^ cells within CD8^+^ T cells (D), γδ T cells (TCRγδ^+^) and NKT cells (NK1.1^+^CD3^+^) (E), and monocytes (CD11b^+^CD11c^−^), dendritic cells (CD11c^+^) and NK cells (NKp46^+^) (F), in the blood and spleen of WT and APPPS1 mice treated with either IgG1 or anti-CD25 antibody. Mean +/− SEM (from *n* = 2–4 mice/ group). Mann–Whitney test: *P < 0.05; **P < 0.01; ***P < 0.001.

**Additional file 6: Figure S6.** Amplification of Tregs does not consistently affect other peripheral immune populations. Percentage of B cells (CD19^+^) and T cells (CD3^+^) (A), CD4^+^ and CD8^+^ T cells within CD3^+^ cells (B), Th1 (T-bet^+^), Th17 (RORγT^+^) and Th2 (GATA3^+^) within CD4^+^ T cells (C), CD25^+^Foxp3^+^ and CD25^+^Foxp3^−^ cells within CD8^+^ T cells (D), γδ T cells (TCRγδ^+^) and NKT cells (NK1.1^+^CD3^+^) (E), and monocytes (CD11b^+^CD11c^−^), dendritic cells (CD11c^+^) and NK cells (NKp46^+^) (F), in the blood and spleen of WT and APPPS1 mice treated with either PBS or low-dose IL-2. Mean +/− SEM (from *n* = 3–5 mice/ group). Mann–Whitney test: *P < 0.05; **P < 0.01; ***P < 0.001.

**Additional file 7: Figure S7.** Modulation of Tregs does not consistently affect the ratio of naive and antigen-experienced Tregs. **A.** Percentage of CD4^+^CD25^+^Foxp3^+^ Tregs within total CD4^+^ T cells in the blood of WT and APPPS1 mice. **B.** Percentage of naive (CD62L^+^CD44^−^) and activated/memory (CD62L^−^CD44^+^) Tregs within CD4^+^CD25^+^Foxp3^+^ Tregs, in the blood of WT and APPPS1 mice. **C-D.** Percentage of naive (CD62L^+^CD44^−^) and activated/memory (CD62L^−^CD44^+^) Tregs within CD4^+^CD25^+^Foxp3^+^ Tregs, in the blood of WT and APPPS1 mice treated with either PBS or anti-CD25 antibody (C) or with either PBS or low-dose IL-2 (D). Mean +/− SEM (fn = 2–8 mice/ group). Mann–Whitney test: *P < 0.05; **P < 0.01; ***P < 0.001.

**Additional file 8: Table S1.** RT-qPCR primer sequences list.

## Data Availability

The datasets used and/or analyzed during the current study are available from the corresponding author upon reasonable request.

## Abbreviations

Aβ: Amyloid-beta peptide
AD: Alzheimer’s disease
AMIGO2: Adhesion molecule with Ig-like domain 2
C1qA: Complement C1q A chain
C3: Complement component 3
Cox2: Cyclo-oxygenase 2
DAAs: Disease-associated astrocytes
FKBP5: FKBP prolyl isomerase 5
GFAP: Glial fibrillary acidic protein
SERPING1: Serpin family G member 1
TM4SF1: Transmembrane 4 L six family member 1
TNFα: Tumor necrosis factor α
Tregs: Regulatory T cells

## References

[1] Leng F, Edison P (2021). Neuroinflammation and microglial activation in Alzheimer disease: where do we go from here ?. Nat Rev Neurol.

[2] Krasemann S, Madore C, Cialic R, Baufeld C, Calcagno N, El Fatimy R (2017). The TREM2-APOE pathway drives the transcriptional phenotype of dysfunctional microglia in neurodegenerative diseases. Immunity.

[3] Keren-Shaul H, Spinrad A, Weiner A, Matcovitch-Natan O, Dvir-Szternfeld R, Ulland TK (2017). A unique microglia type associated with restricting development of Alzheimer’s disease. Cell.

[4] Sala Frigerio C, Wolfs L, Fattorelli N, Thrupp N, Voytyuk I, Schmidt I (2019). The major risk factors for Alzheimer’s disease: age, sex, and genes modulate the microglia response to Aβ plaques. Cell Rep.

[5] Seifert G, Schilling K, Steinhäuser C (2006). Astrocyte dysfunction in neurological disorders: a molecular perspective. Nat Rev Neurosci.

[6] Linnerbauer M, Wheeler MA, Quintana FJ (2020). Astrocyte crosstalk in CNS inflammation. Neuron.

[7] Ben Haim L, Carrillo-de Sauvage MA, Ceyzériat K, Escartin C (2015). Elusive roles for reactive astrocytes in neurodegenerative diseases. Front Cell Neurosci.

[8] Sofroniew MV, Vinters HV (2010). Astrocytes: biology and pathology. Acta Neuropathol.

[9] Anderson MA, Ao Y, Sofroniew MV (2014). Heterogeneity of reactive astrocytes. Neurosci Lett.

[10] Liddelow SA, Guttenplan KA, Clarke LE, Bennett FC, Bohlen CJ, Schirmer L (2017). Neurotoxic reactive astrocytes are induced by activated microglia. Nature.

[11] King A, Szekely B, Calapkulu E, Ali H, Rios F, Jones S (2020). The increased densities, but different distributions, of both C3 and S100A10 immunopositive astrocyte-like cells in Alzheimer’s disease brains suggest possible roles for both A1 and A2 astrocytes in the disease pathogenesis. Brain Sci.

[12] Kraft AW, Hu X, Yoon H, Yan P, Xiao Q, Wang Y (2013). Attenuating astrocyte activation accelerates plaque pathogenesis in APP/PS1 mice. FASEB J.

[13] Shi Q, Chowdhury S, Ma R, Le KX, Hong S, Caldarone BJ (2017). Complement C3 deficiency protects against neurodegeneration in aged plaque-rich APP/PS1 mice. Sci Transl Med..

[14] Goetzl EJ, Schwartz JB, Abner EL, Jicha GA, Kapogiannis D (2018). High complement levels in astrocyte-derived exosomes of Alzheimer disease. Ann Neurol.

[15] Rosset BM, Lui G, Dansokho C, Chaigneau T, Dorothée G (2015). Vaccine-induced Aβ-specific CD8+ T cells do not trigger autoimmune neuroinflammation in a murine model of Alzheimer’s disease. J Neuroinflammation.

[16] Monsonego A, Imitola J, Petrovic S, Zota V, Nemirovsky A, Baron R (2006). Aβ-induced meningoencephalitis is IFN-γ-dependent and is associated with T cell-dependent clearance of Aβ in a mouse model of Alzheimer’s disease. Proc Natl Acad Sci U S A.

[17] Browne TC, McQuillan K, McManus RM, O’Reilly J-A, Mills KHG, Lynch MA (2013). IFN-γ production by amyloid β-specific Th1 cells promotes microglial activation and increases plaque burden in a mouse model of Alzheimer’s disease. J Immunol.

[18] Cao C, Arendash GW, Dickson A, Mamcarz MB, Lin X, Ethell DW (2009). Aβ-specific Th2 cells provide cognitive and pathological benefits to Alzheimer’s mice without infiltrating the CNS. Neurobiol Dis.

[19] Ethell DW, Shippy D, Cao C, Cracchiolo JR, Runfeldt M, Blake B (2006). Aβ-specific T-cells reverse cognitive decline and synaptic loss in Alzheimer’s mice. Neurobiol Dis.

[20] Laurent C, Dorothée G, Hunot S, Martin E, Monnet Y, Duchamp M (2017). Hippocampal T cell infiltration promotes neuroinflammation and cognitive decline in a mouse model of tauopathy. Brain.

[21] Dansokho C, Ait Ahmed D, Aid S, Toly-Ndour C, Chaigneau T, Calle V (2016). Regulatory T cells delay disease progression in Alzheimer-like pathology. Brain.

[22] Baek H, Ye M, Kang GH, Lee C, Lee G, Bin CD (2016). Neuroprotective effects of CD4+CD25+Foxp3+ regulatory T cells in a 3xTg-AD Alzheimer’s disease model. Oncotarget.

[23] Alves S, Churlaud G, Audrain M, Michaelsen-Preusse K, Fol R, Souchet B (2017). Interleukin-2 improves amyloid pathology, synaptic failure and memory in Alzheimer’s disease mice. Brain.

[24] Guttenplan KA, Weigel MK, Prakash P, Wijewardhane PR, Hasel P, Rufen-Blanchette U (2021). Neurotoxic reactive astrocytes induce cell death via saturated lipids. Nature.

[25] Litvinchuk A, Wan YW, Swartzlander DB, Chen F, Cole A, Propson NE (2018). Complement C3aR inactivation attenuates tau pathology and reverses an immune network deregulated in tauopathy models and Alzheimer’s disease. Neuron.

[26] Lian H, Yang L, Cole A, Sun L, Chiang ACA, Fowler SW (2015). NFκB-activated astroglial release of complement C3 compromises neuronal morphology and function associated with Alzheimer’s disease. Neuron.

[27] Stevens B, Allen NJ, Vazquez LE, Howell GR, Christopherson KS, Nouri N (2007). The classical complement cascade mediates CNS synapse elimination. Cell.

[28] Hong S, Beja-Glasser VF, Nfonoyim BM, Frouin A, Li S, Ramakrishnan S (2016). Complement and microglia mediate early synapse loss in Alzheimer mouse models. Science.

[29] Baruch K, Rosenzweig N, Kertser A, Deczkowska A, Sharif AM, Spinrad A (2015). Breaking immune tolerance by targeting Foxp3+ regulatory T cells mitigates Alzheimer’s disease pathology. Nat Commun.

[30] Clarke LE, Liddelow SA, Chakraborty C, Münch AE, Heiman M, Barres BA (2018). Normal aging induces A1-like astrocyte reactivity. Proc Natl Acad Sci U S A.

[31] Xu X, Zhang A, Zhu Y, He W, Di W, Fang Y (2018). MFG-E8 reverses microglial-induced neurotoxic astrocyte (A1) via NF-κB and PI3K-Akt pathways. J Cell Physiol.

[32] Hasel P, Rose IVL, Sadick JS, Kim RD, Liddelow SA (2021). Neuroinflammatory astrocyte subtypes in the mouse brain. Nat Neurosci.

[33] Sadick JS, O’Dea MR, Hasel P, Dykstra T, Faustin A, Liddelow SA (2022). Astrocytes and oligodendrocytes undergo subtype-specific transcriptional changes in Alzheimer’s disease. Neuron.

[34] Jiwaji Z, Tiwari SS, Avilés-Reyes RX, Hooley M, Hampton D, Torvell M (2022). Reactive astrocytes acquire neuroprotective as well as deleterious signatures in response to Tau and Aß pathology. Nat Commun.

[35] Ceyzériat K, Ben Haim L, Denizot A, Pommier D, Matos M, Guillemaud O (2018). Modulation of astrocyte reactivity improves functional deficits in mouse models of Alzheimer’s disease. Acta Neuropathol Commun.

[36] Habib N, McCabe C, Medina S, Varshavsky M, Kitsberg D, Dvir-Szternfeld R (2020). Disease-associated astrocytes in Alzheimer’s disease and aging. Nat Neurosci.

[37] Escartin C, Galea E, Lakatos A, O’Callaghan JP, Petzold GC, Serrano-Pozo A (2021). Reactive astrocyte nomenclature, definitions, and future directions. Nat Neurosci.

[38] Yu X, Nagai J, Khakh BS (2020). Improved tools to study astrocytes. Nat Rev Neurosci.

[39] Wilhelmsson U, Bushong EA, Price DL, Smarr BL, Phung V, Terada M (2006). Redefining the concept of reactive astrocytes as cells that remain within their unique domains upon reaction to injury. Proc Natl Acad Sci U S A.

