# Supplementary figures and images for: Regulatory T cells decrease C3-positive reactive astrocytes in Alzheimer-like pathology
Source: J Neuroinflammation. 2023 Mar 8;20:64. doi: 10.1186/s12974-023-02702-3 (PMC9996941; doi:10.1186/s12974-023-02702-3)

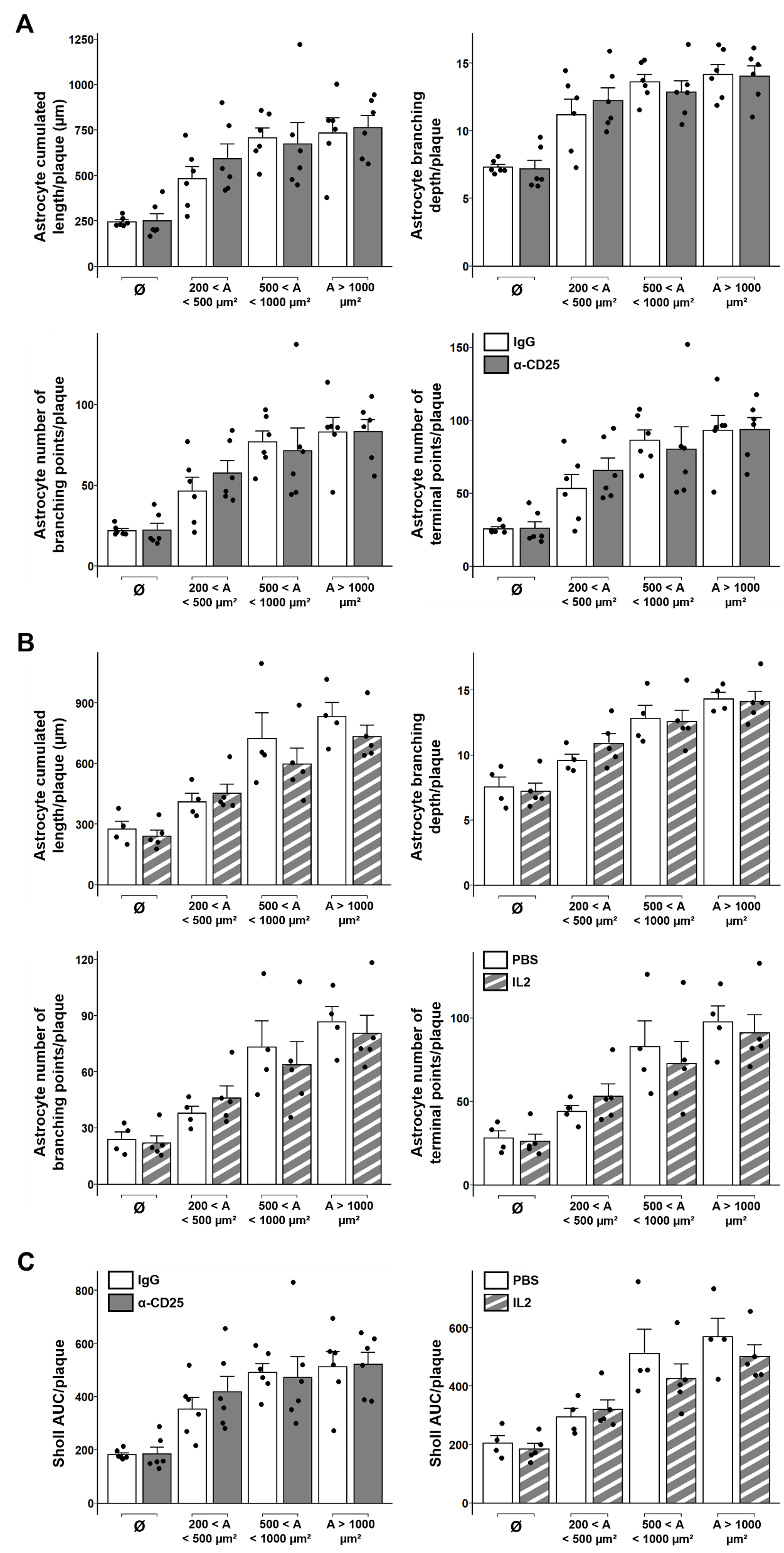

Supplement: Supplementary file 2 — Additional file 2: Figure S2. Modulation of Tregs does not affect the morphology and branching complexity of plaque-associated astrocytes. A-B. Analysis of astrocytes branching complexity in the cerebral cortex of APPPS1 mice treated for either the depletion (A) or amplification of Tregs (B). Quantification of i) astrocytes processes cumulated length (top-left), ii) astrocytes branching depth index (top-right), iii) astrocytes number of processes branching points (bottom-left) and iv) astrocytes number of processes terminal points (bottom-right) in close vicinity to amyloid deposits of different size ranges are shown for each treatment. C. Mean area under curve of the Sholl analysis presented in Fig. 3C on APPPS1 mice treated for either the depletion or the amplification of Tregs. Results are represented according to the size of amyloid deposits. Unrecruited astrocytes are referenced as Ø. Mean +/− SEM (n > 30 amyloid deposits from 4–6 mice/ group). Mann–Whitney test: *P < 0.05; **P < 0.01; ***P < 0.001. [file 12974_2023_2702_MOESM2_ESM.tif]

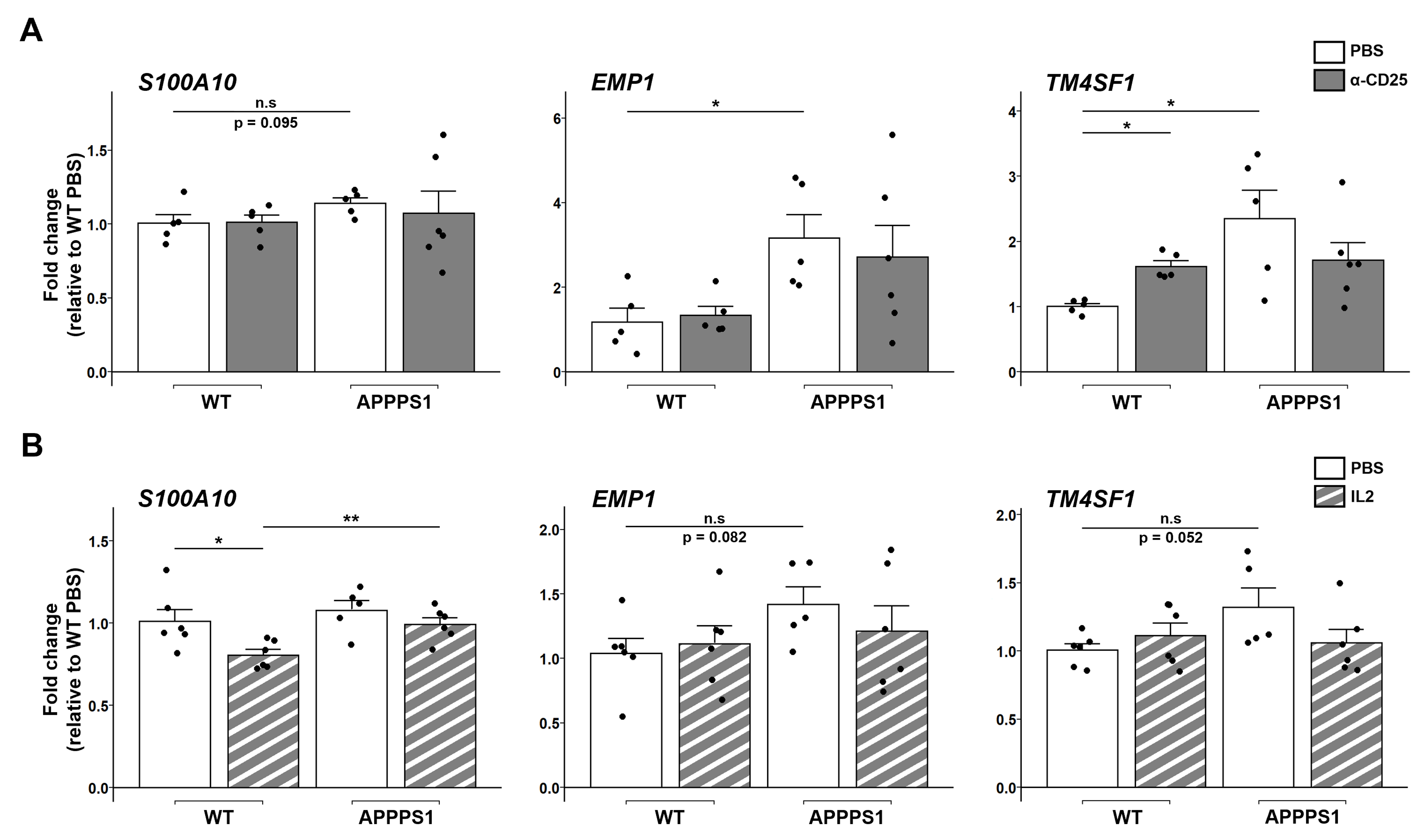

Supplement: Supplementary file 3 — Additional file 3: Figure S3. Modulation of Tregs does not consistently affect the gene expression of several A2-like astrocytic markers. A-B. Quantification of mRNA coding for the A2-like astrocytic markers S100A10, EMP1 and TM4SF1 in the brain of mice treated with either PBS or anti-CD25 antibody (A) or with either PBS or IL-2 (B). Values were first normalized according to the expression of the housekeeping gene PPIA and then normalized to the mean value of the PBS-injected WT control group. Mean +/− SEM (from n = 5–6 mice/ group). Mann–Whitney test: *P < 0.05; **P < 0.01; ***P < 0.001. [file 12974_2023_2702_MOESM3_ESM.tif]

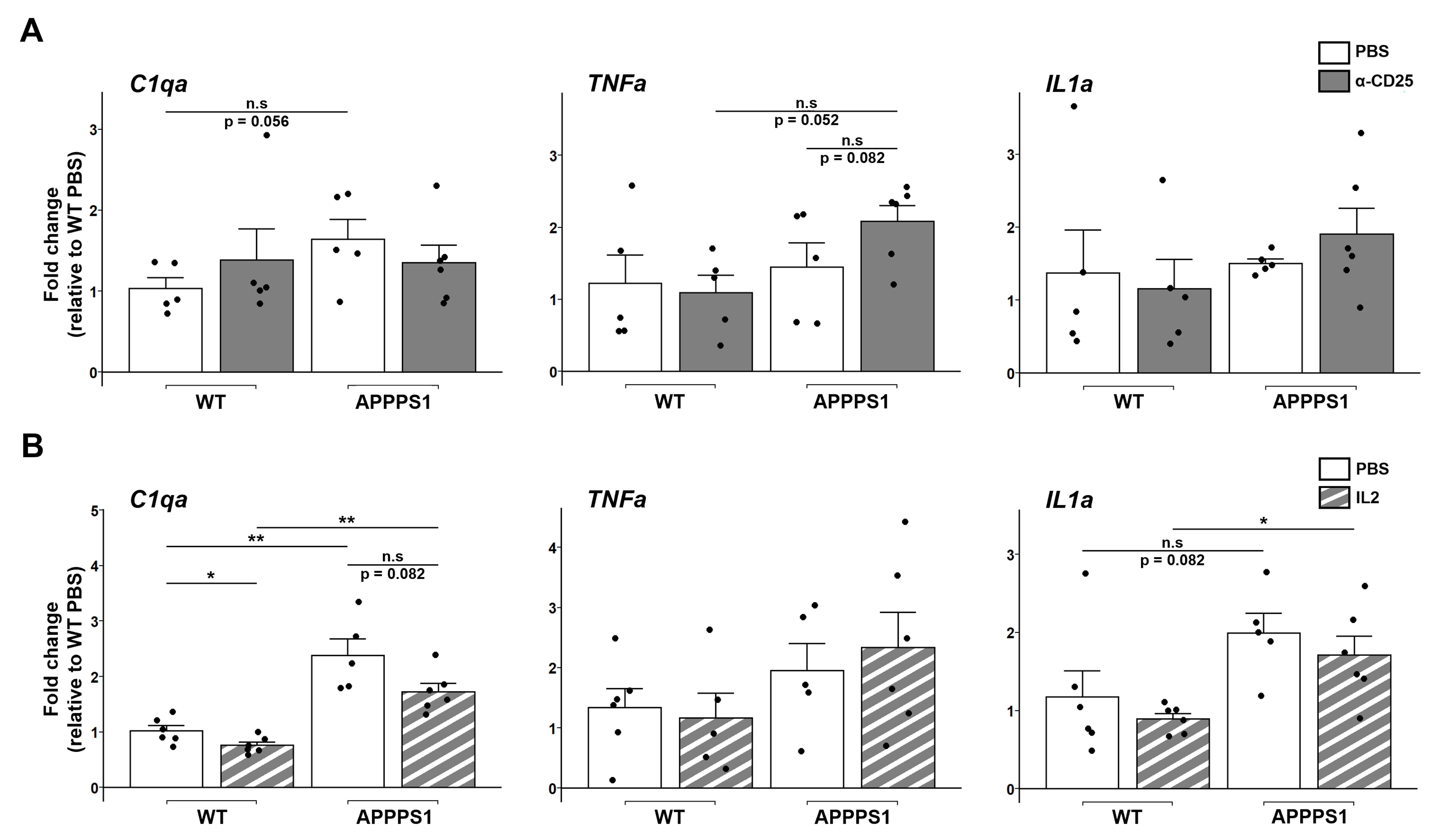

Supplement: Supplementary file 4 — Additional file 4: Figure S4. Modulation of Tregs does not consistently affect the expression of several A1-like reactive astrocyte polarization factors. A-B. Quantification of mRNA coding for the A1-like reactive astrocyte polarization factors C1qa, TNFα, and IL-1α in the brain of mice treated with either PBS or anti-CD25 antibody (A) or with either PBS or IL-2 (B). Values were first normalized according to the expression of the housekeeping gene PPIA and then normalized to the mean value of the PBS-injected WT control group. Mean +/− SEM (from n = 5–6 mice/ group). Mann–Whitney test: *P < 0.05; **P < 0.01; ***P < 0.001. [file 12974_2023_2702_MOESM4_ESM.tif]

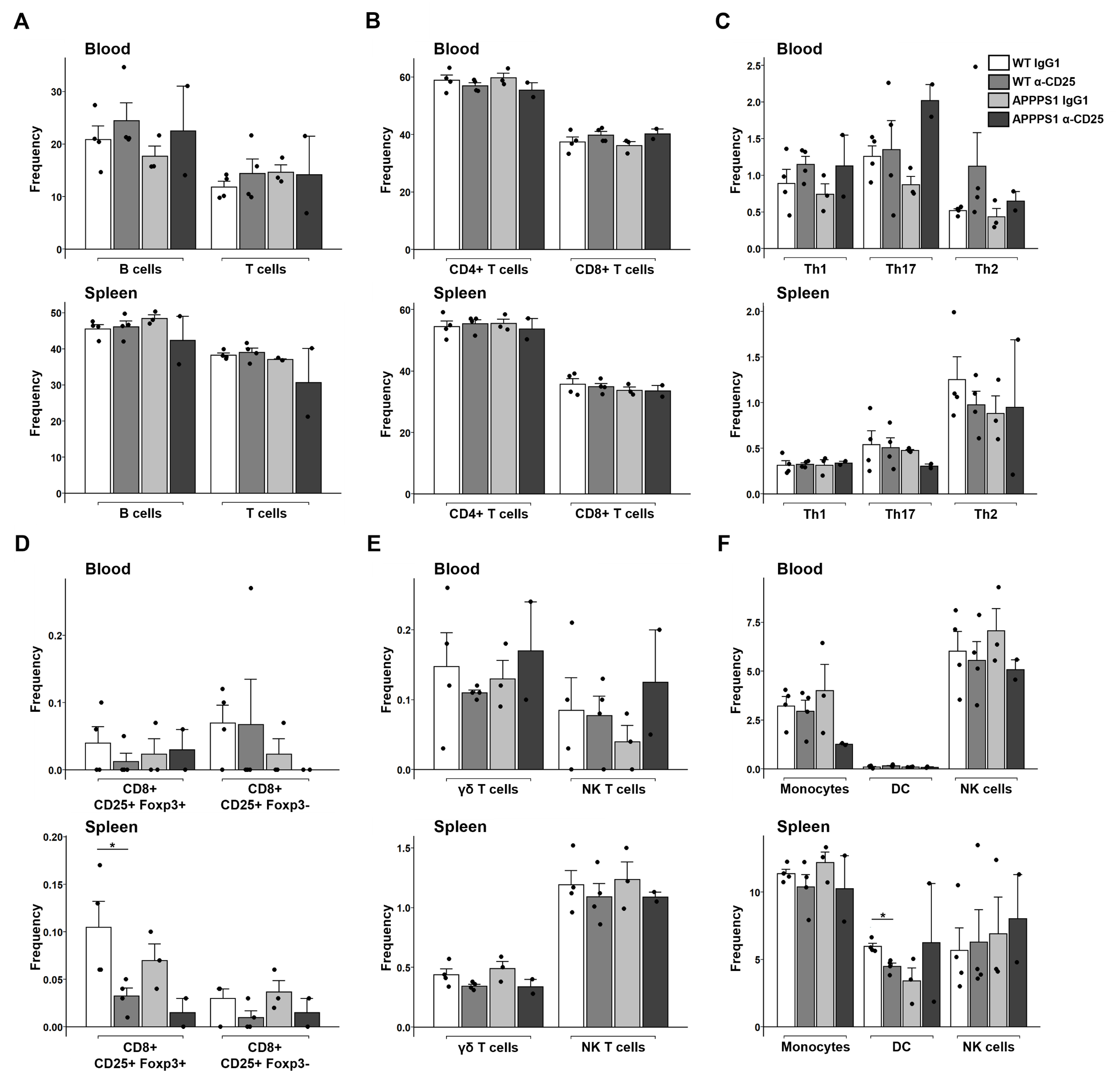

Supplement: Supplementary file 5 — Additional file 5: Figure S5. Depletion of Tregs does not consistently affect other peripheral immune populations. Percentage of B cells (CD19+) and T cells (CD3+) (A), CD4+ and CD8+ T cells within CD3+ cells (B), Th1 (T-bet+), Th17 (RORγT+) and Th2 (GATA3+) within CD4+ T cells (C), CD25+Foxp3+ and CD25+Foxp3− cells within CD8+ T cells (D), γδ T cells (TCRγδ+) and NKT cells (NK1.1+CD3+) (E), and monocytes (CD11b+CD11c−), dendritic cells (CD11c+) and NK cells (NKp46+) (F), in the blood and spleen of WT and APPPS1 mice treated with either IgG1 or anti-CD25 antibody. Mean +/− SEM (from n = 2–4 mice/ group). Mann–Whitney test: *P < 0.05; **P < 0.01; ***P < 0.001. [file 12974_2023_2702_MOESM5_ESM.tif]

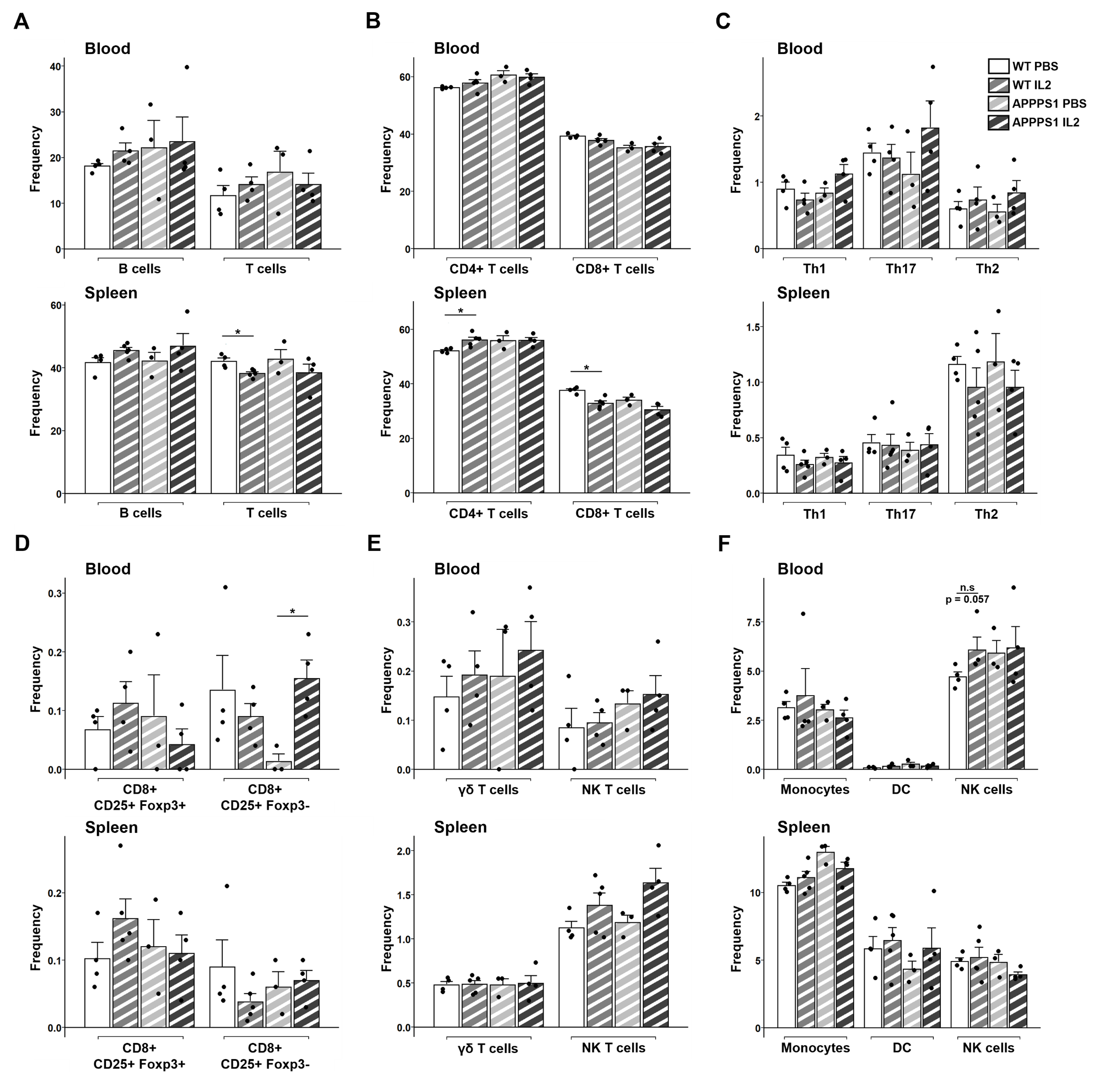

Supplement: Supplementary file 6 — Additional file 6: Figure S6. Amplification of Tregs does not consistently affect other peripheral immune populations. Percentage of B cells (CD19+) and T cells (CD3+) (A), CD4+ and CD8+ T cells within CD3+ cells (B), Th1 (T-bet+), Th17 (RORγT+) and Th2 (GATA3+) within CD4+ T cells (C), CD25+Foxp3+ and CD25+Foxp3− cells within CD8+ T cells (D), γδ T cells (TCRγδ+) and NKT cells (NK1.1+CD3+) (E), and monocytes (CD11b+CD11c−), dendritic cells (CD11c+) and NK cells (NKp46+) (F), in the blood and spleen of WT and APPPS1 mice treated with either PBS or low-dose IL-2. Mean +/− SEM (from n = 3–5 mice/ group). Mann–Whitney test: *P < 0.05; **P < 0.01; ***P < 0.001. [file 12974_2023_2702_MOESM6_ESM.tif]
