# Supplementary material for: Regulatory T cells decrease C3-positive reactive astrocytes in Alzheimer-like pathology
Source: J Neuroinflammation. 2023 Mar 8;20:64. doi: 10.1186/s12974-023-02702-3 (PMC9996941; doi:10.1186/s12974-023-02702-3)
Supplement: Supplementary file 8 — Additional file 8: Table S1. RT-qPCR primer sequences list. [file 12974_2023_2702_MOESM8_ESM.docx]

**Table S1: RT-qPCR primer sequences list.**

| **Gene** | **Forward (5' - 3')** | **Reverse (3' - 5')** |
| --- | --- | --- |
| **HPRT** | TGACACTGGCAAAACAATGCA | GGTCCTTTTCACCAGCAAGCT |
| **PPIA** | GGCAAATGCTGGACCAAAC | CATTCCTGGACCCAAAACG |
| **GFAP** | AGAAAGGTTGAATCGCTGGA | CGGCGATAGTCGTTAGCTTC |
| **ASPG** | GCTGCTGGCCATTTACACTG | GTGGGCCTGTGCATACTCTT |
| **OSMR** | GTGAAGGACCCAAAGCATGT | GCCTAATACCTGGTGCGTGT |
| **SERPINA3N** | CAACCAGAGACCCTGAGGAAGT | AGGACATCCTCCAGGCTGTAGT |
| **ALDH1L1** | GCAGGTACTTCTGGGTTGCT | GGAAGGCACCCAAGGTCAAA |
| **SERPING1** | ACAGCCCCCTCTGAATTCTT | GGATGCTCTCCAAGTTGCTC |
| **H2D1** | TCCGAGATTGTAAAGCGTGAAGA | ACAGGGCAGTGCAGGGATAG |
| **FKBP5** | TATGCTTATGGCTCGGCTGG | CAGCCTTCCAGGTGGACTTT |
| **AMIGO2** | GAGGCGACCATAATGTCGTT | GCATCCAACAGTCCGATTCT |
| **S100A10** | CCTCTGGCTGTGGACAAAAT | CTGCTCACAAGAAGCAGTGG |
| **TM4SF1** | GCCCAAGCATATTGTGGAGT | AGGGTAGGATGTGGCACAAG |
| **EMP1** | GAGACACTGGCCAGAAAAGC | TAAAAGGCAAGGGAATGCAC |
| **C1qA** | GGAGCATCCAGTTTGATCG | CATCCCTGAGAGGTCTCCAT |
| **IL1α** | TCTCAGATTCACAACTGTTCGTG | AGAAAATGAGGTCGGTCTCACTA |
| **TNFα** | GTCTACTGAACTTCGGGGTGAT | ATGATCTGAGTGTGAGGGTCTG |
